# Cross-species transmission of an ancient endogenous retrovirus and convergent co-option of its envelope gene in two mammalian orders

**DOI:** 10.1371/journal.pgen.1010458

**Published:** 2022-10-14

**Authors:** J’Zaria Simpson, Christine A. Kozak, Guney Boso

**Affiliations:** Laboratory of Molecular Microbiology, National Institute of Allergy and Infectious Diseases, Bethesda, Maryland, United States of America; National Institute of Genetics, JAPAN

## Abstract

Endogenous retroviruses (ERVs) found in vertebrate genomes are remnants of retroviral invasions of their ancestral species. ERVs thus represent molecular fossil records of ancient retroviruses and provide a unique opportunity to study viral-host interactions, including cross-species transmissions, in deep time. While most ERVs contain the mutated remains of the original retrovirus, on rare occasions evolutionary selection pressures lead to the co-option/exaptation of ERV genes for a host function. Here, we report the identification of two ancient related non-orthologous ERV *env* genes, *ARTenvV* and *CARenvV*, that are preserved with large open reading frames (ORFs) in the mammalian orders Artiodactyla and Carnivora, respectively, but are not found in other mammals. These Env proteins lack a transmembrane motif, but phylogenetic analyses show strong sequence preservation and positive selection of the *env* surface ORF in their respective orders, and transcriptomic analyses show a broad tissue expression pattern for both *ARTenvV* and *CARenvV*, suggesting that these genes may be exapted for a host function. Multiple lines of evidence indicate that *ARTenvV* and *CARenvV* were derived from an ancient ancestral exogenous gamma-like retrovirus that was independently endogenized in two mammalian orders more than 60 million years ago, which roughly coincides with the K-Pg mass extinction event and subsequent mammalian diversification. Thus, these findings identify the oldest known retroviral cross-ordinal transmission of a gamma-like retrovirus with no known extant infectious counterpart in mammals, and the first discovery of the convergent co-option of an ERV gene derived from the same ancestral retrovirus in two different mammalian orders.

## Author summary

A unique feature of retrovirus replication results in the integration of the viral genome into host chromosomes. These insertions can be preserved as part of the host genome. These relics of infections by ancient retroviruses are termed endogenous retroviruses (ERVs). In this study, we identified two independently acquired ERVs in the genomes of even-toed ungulates and carnivores both of which contain related retroviral envelope genes. Phylogenetic analyses and the broad tissue expression of these genes suggest that they may be domesticated for a host function. Analyses of their sequence and genomic locations indicate that these ERVs were acquired by the ancestors of these two mammalian orders as a result of an ancient cross-species transmission event at least 60 million years ago. These findings identify the oldest cross-ordinal retroviral transmission event in mammals and the first evidence of independent domestication of an envelope gene from the same parental retrovirus.

## Introduction

The retroviral replication cycle includes an obligatory step that is unique among animal viruses in which the reverse transcribed DNA copy of the viral RNA genome is integrated into host chromosomes and becomes a permanent part of the genomic DNA of the infected cell [1]. While exogenous retroviruses spread via horizontal transmission between individuals following the infection of somatic cells, when a retrovirus infects a germline cell or one of its precursors, this provirus can be vertically transmitted to the progeny of that individual [2,3]. These endogenous retroviruses (ERVs) make up a significant portion of vertebrate genomes (E.g., 8–10% of the human and mouse genomes) [4,5]. Despite being derived from rapidly evolving viruses, once fixed in the genome of a host, ERVs are subject to genetic drift and the neutral mutation rate of the host species. Over time, without selective forces to keep ERVs intact, but in the face of evolutionary pressures to remove or disable them, the vast majority of ERVs undergo successive deletions and other mutations leaving behind only remnants that identify the original exogenous retrovirus. Comparative genomics has shown that the ERV remains found in vertebrate genomes represent valuable molecular “fossil” records of ancient retroviruses and offer a window into the history of extinct retroviruses and their ancient hosts in deep time [6–10].

Cross-species transmission of viruses presents a significant threat to both human and animal health. Most human viral diseases are caused by zoonotic viruses that have jumped the species barrier to infect humans including rabies, influenza, Nipah, SARS and Ebola viruses [11–16]. In the case of retroviruses, the pandemic caused by HIV-1 group M arose following the transmission of SIVcpz from chimpanzees to humans in the early 20^th^ century [17–19]. Similar cross-species transmission events are thought to have occurred between humans and various primates leading to the emergence of other disease-causing retroviruses in humans including HIV-2 and HTLVs [20–22]. Although the known infectious human retroviruses have jumped from closely related primate species [17,20,21,23], retroviral cross-species transmission events between other mammalian species have been reported and some of these jumps have resulted in endogenization [24–27]. ERVs thus provide a unique opportunity to study cross-species transmissions that occurred during the evolution of mammals [8,28–30].

While most mammalian ERVs are mutationally degraded or truncated, on rare occasions, when an ERV gene is beneficial to the host, it can be co-opted for a physiological function with evolutionary pressures keeping it intact for millions of years [3]. The most well-documented examples of this phenomenon are the fusogenic ERV envelope-derived genes in mammals called syncytins that are highly expressed in placental syncytiotrophoblasts and function in the formation of this temporary organ at the materno-fetal interface [31–33]. The various mammalian species rely on different syncytins, all of which were co-opted millions of years ago from different ERV insertions representing an extraordinary example of convergent evolution [34–42]. In addition to syncytins, a number of other ERV-derived genes have been co-opted for antiviral as well as other functions, including the retroviral restriction factor *Fv1* of rodents which was derived from the *gag* gene of an ancient ERV-L-like element [43–45] and a truncated ERV envelope gene called suppressyn found in primates that is proposed to interfere with the binding of syncytin to its receptor [46].

We recently described an ERV-derived *gag* gene, *gagV1*, in the human genome that is specifically expressed in the placenta and conserved in all lineages of simian primates [47]. The provirus that contains *gagV1*, HERV-V1, also encodes an *env* gene called *envV1* [47–49]. This region of the human genome contains an additional related provirus, HERV-V2, with a highly similar, intact *env* gene called *envV2* [47–49]. In the present study, we report the discovery of two ERV *env* genes that are related to primate *envV1* and *envV2* in the mammalian orders Artiodactyla (even-toed ungulates) and Carnivora. Phylogenomic analyses show that these non-orthologous *env* genes, here named *ARTenvV* and *CARenvV*, contain highly conserved ORFs in all lineages of their respective orders and are part of ERVs that became independently fixed in the genomes of the common ancestors of extant members of Artiodactyla and Carnivora. Both *ARTenvV* and *CARenvV* produce transcripts derived from the splicing of two exons and are expressed in a variety of tissues suggesting that they are co-opted for a host function. While *ARTenvV* and *CARenvV* ORFs show high sequence conservation in their respective orders, we also found evidence of positive selection in a few codons of both of these genes. Despite being separated by more than 60 million years of evolution, nucleotide sequences of *ARTenvV* and *CARenvV* show remarkably high similarity in the surface (SU) subunit of the *env* gene suggesting that they were derived from the same exogenous retrovirus in an extraordinary example of an ancient cross-species transmission event.

## Results

### A conserved *env* ORF in Carnivora related to the HERV genes *envV1* and *envV2*

We performed an *in silico* search of the annotated mammalian genomes to identify ERVs related to HERV-V using tBLASTn. As expected, the vast majority of the hits are the *envV1* and *envV2* primate orthologs. Outside primates, we found three hits in the Ursidae family (bears) in the order Carnivora that showed up to 45% identity to EnvV2. An iterative BLAST search of other Carnivora species using the DNA sequence of the Ursidae hits revealed highly similar sequences in the rest of the Carnivora species.

**Fig 1:**
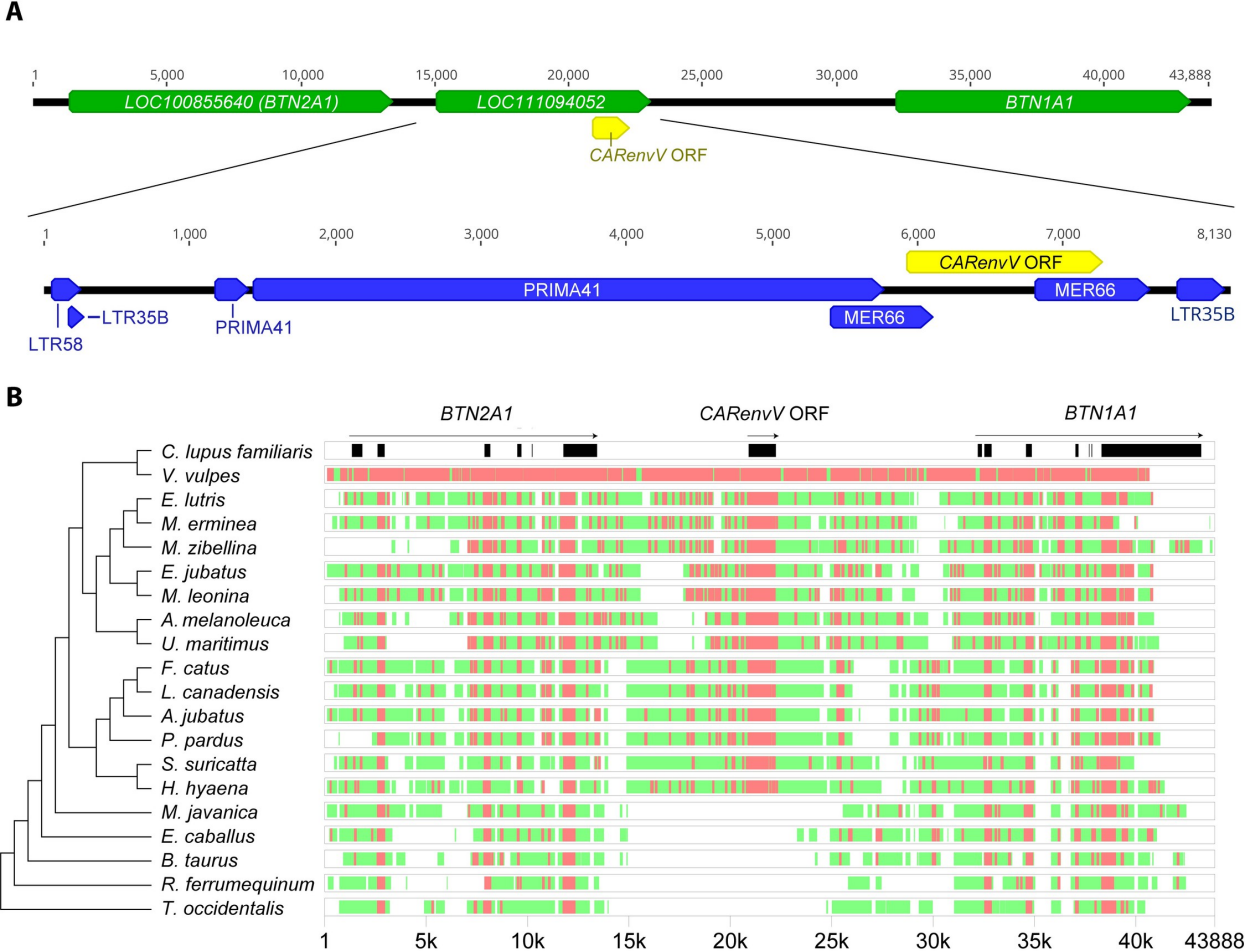
Genomic location and conserved synteny of *CARenvV*. **A.** (Upper panel) Chromosomal context of the *CARenvV* locus is shown for the *C*. *lupus familiaris* (domestic dog) genome (ROS_Cfam_1.0, chromosome 35). Refseq annotated genes are shown in green, and the *CARenvV* ORF is in yellow. (Lower panel) ERV derived repeat elements identified by the Dfam repeat database for the indicated region are annotated in blue. **B.** Genomic segments containing the *CARenvV* ORF and the *BTN1A1* and *BTN2A1* genes were extracted from the NCBI genome database for each of the indicated species and aligned using the MultiPipMaker alignment tool [108]. The *CARenvV* ORF and the *BTN1A1* and *BTN2A1* exons are shown in black boxes in the *C*. *lupus familiaris* reference assembly. For the other species, regions with more than 75% identity are shown as red boxes while regions with 50–75% identity are shown as green boxes. On the left is a cladogram representing the phylogenetic relationships for the species analyzed that was generated using TimeTree [82].

A closer look at the genomic location of this ERV in the domestic dog (*Canis lupus familiaris*) positions it between the genes *BTN1A1* and *BTN2A1* (Fig 1A). There is an open reading frame (ORF) of 1344 bp towards the 3’ end of this ERV that is upstream of the putative 3’ LTR (Fig 1A). A search of the PANTHER protein domain database [50] showed that the putative protein encoded by this ORF is closely related to the retroviral envelope protein subfamily (PTHR10424:SF74) that includes EnvV1 and EnvV2 [51]. This *env* ORF is also predicted to be part of a gene and a transcript annotated as “endogenous retrovirus group V member 2 Env polyprotein-like” in the NCBI RefSeq database. Hence, we named this gene *CARenvV*.

Further analysis of *CARenvV* using the Dfam repeat database revealed remnants of *gag* and *pol* genes with similarity to an ERV group named Prima41 (Fig 1A), while its C-terminal portion shows similarity to the ERV group MER66 (Fig 1A) [52].

This provirus also contains remnants of 5’ and 3’ LTRs (Fig 1A) [52]. Since retroviral insertion into the host genome generates identical 5’ and 3’ LTRs, the divergence between the LTRs of an ERV can be used to estimate its age [53,54]. Because the *CARenvV* ERV in *C*. *lupus familiaris* contains a partial deletion of the 5’ LTR, we used the *CARenvV* ortholog of *F*. *catus* for this analysis. Comparison of these 5’ and 3’ LTRs (S1 File) determined this ERV to be ancient (91–121 million years old) putting its endogenization before the divergence of placental mammals [55,56].

**Fig 2:**
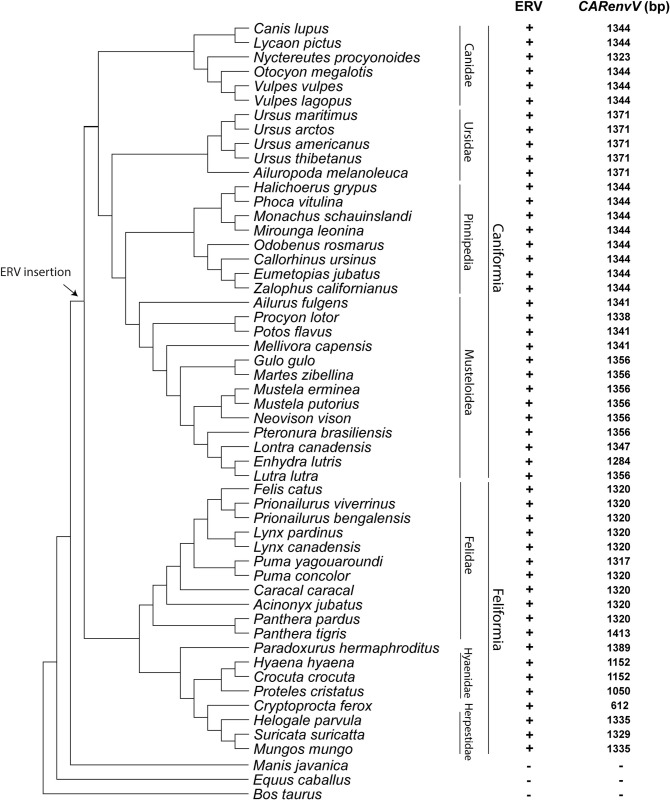
Conservation of *CARenvV* ORF in the lineages of Carnivora. A cladogram illustrates the evolutionary relationships of species with assembled genomes in the order Carnivora along with three species from sister orders of Carnivora. The tree was generated with TimeTree [82]. Suborder and family classifications are shown on the right. + indicates the presence and - indicates the absence of ERV orthologs that contain *CARenvV* as identified via BLAST searches of each genome assembly. The size of the *CARenvV* ORF found in the orthologous location at each genome assembly is indicated. An arrow marks the likely insertion and fixation of the ERV that contains *CARenvV*, based on the orthology analysis.

The age of an ERV can also be estimated from the orthology of its genomic location since retroviral integration is not site specific [54,57,58]. We extracted and aligned orthologous segments from the genomes of 15 Carnivora species and from 5 species in sister orders within the superorder Laurasiatheria (S1 Table). We observed high levels of sequence conservation of the *CARenvV* ORF among Carnivora as well as sequence conservation in most exons of the linked *BTN1A1* and *BTN2A1* genes in all the species examined confirming the orthology of this region (Fig 1B). In addition, the region that encompasses the *CARenvV* ORF, as well as the rest of the provirus, is missing in mammalian species outside of Carnivora (Fig 1B). *CARenvV* and its large ORF are conserved in individual Carnivora species in all lineages of this order, although a few species, including three members of the subfamily Hyaenidae, contain early stop codons in the *CARenvV* gene generating shorter ORFs (Fig 2). These results indicate that *CARenvV* was inherited from the common ancestor of the order Carnivora at least 54 mya [59] contradicting the older age estimate from the LTR dating analysis. LTR analyses can produce these older age estimates due to differential selective pressure on ERV LTRs or recombination with similar LTRs. Therefore, these findings collectively indicate that this ancient ERV was fixed in the common ancestor of the extant members of the order Carnivora at least 54 million years ago [59].

### An ancient ERV with an intact SU*env* domain in Artiodactyla

**Fig 3:**
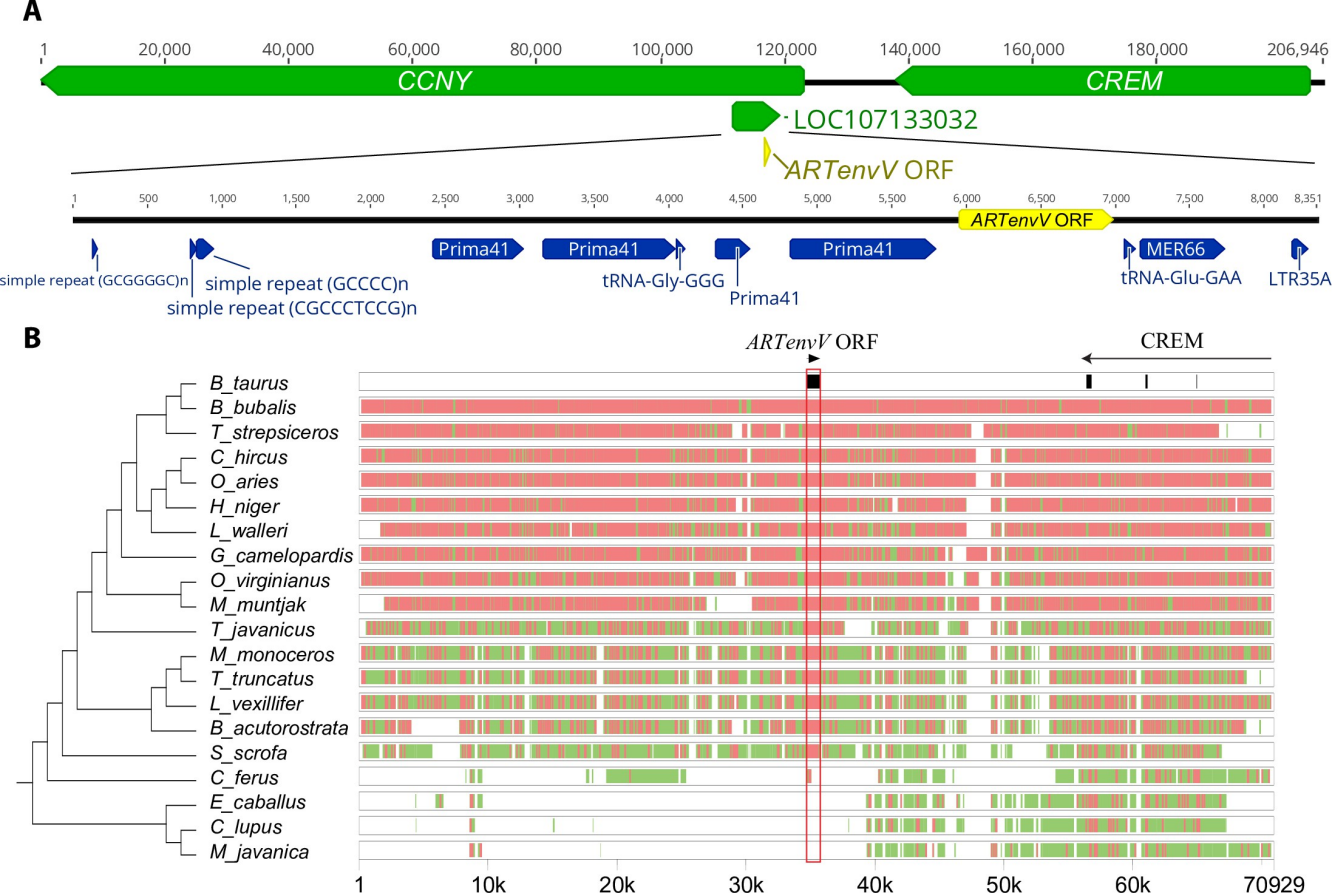
Genomic location and conserved synteny of *ARTenvV*. **A.** (Upper panel) The chromosomal context of the *ARTenvV* locus is shown for *B*. *taurus* (ARS-UCD1.2, chromosome 13). Refseq annotated genes are shown in green with the location of the *ARTenvV* ORF in yellow. (Lower panel) ERV derived repeat elements that are identified by the Dfam repeat database for the indicated region are annotated in blue. **B.** Genomic segments containing *ARTenvV* ORF and the *CREM* gene were extracted from the NCBI genome database for each of the indicated species and aligned using the MultiPipMaker alignment tool [108]. *ARTenvV* ORF and the exons of *CREM* are shown in black boxes in the *B*. *taurus* reference assembly. Regions with more than 75% identity are shown as red boxes while regions with 50–75% identity are shown as green boxes. On the left is a cladogram representing the phylogenetic relationships for the analyzed species generated using TimeTree [82].

Next, we examined whether other mammalian orders contain similar ERVs. To this end, we used the ORF of the *C*. *lupus familiaris CARenvV* as a probe to search other sequenced mammalian genomes. This search revealed a highly similar sequence (>75% identity) in various members of the order Artiodactyla (even-toed ungulates). In *Bos taurus* (cattle) this sequence is located between the genes *CREM* and *CCNY* (Fig 3A) so it is not orthologous to *CARenvV*. Analysis of this ERV using the Dfam repeat database demonstrated that, like *CARenvV*, remnants of the *gag* and *pol* genes show significant similarity to the ERV group Prima41 while the ERV portion immediately upstream of the predicted 3’ LTR showed similarity to the ERV group MER66 (Fig 3A). Also, like *CARenvV*, this ERV contains a large ORF identified by computer annotation as “endogenous retrovirus group V member 1 Env”. Although there is an identifiable LTR at the 3’ end of the ERV there are no remnants of the 5’ LTR as the region immediately upstream of the annotated gene is dominated by GC-rich repeats (Fig 3A). This ERV is therefore related to *CARenvV* but is not orthologous, so we designated this gene *ARTenvV*.

**Fig 4:**
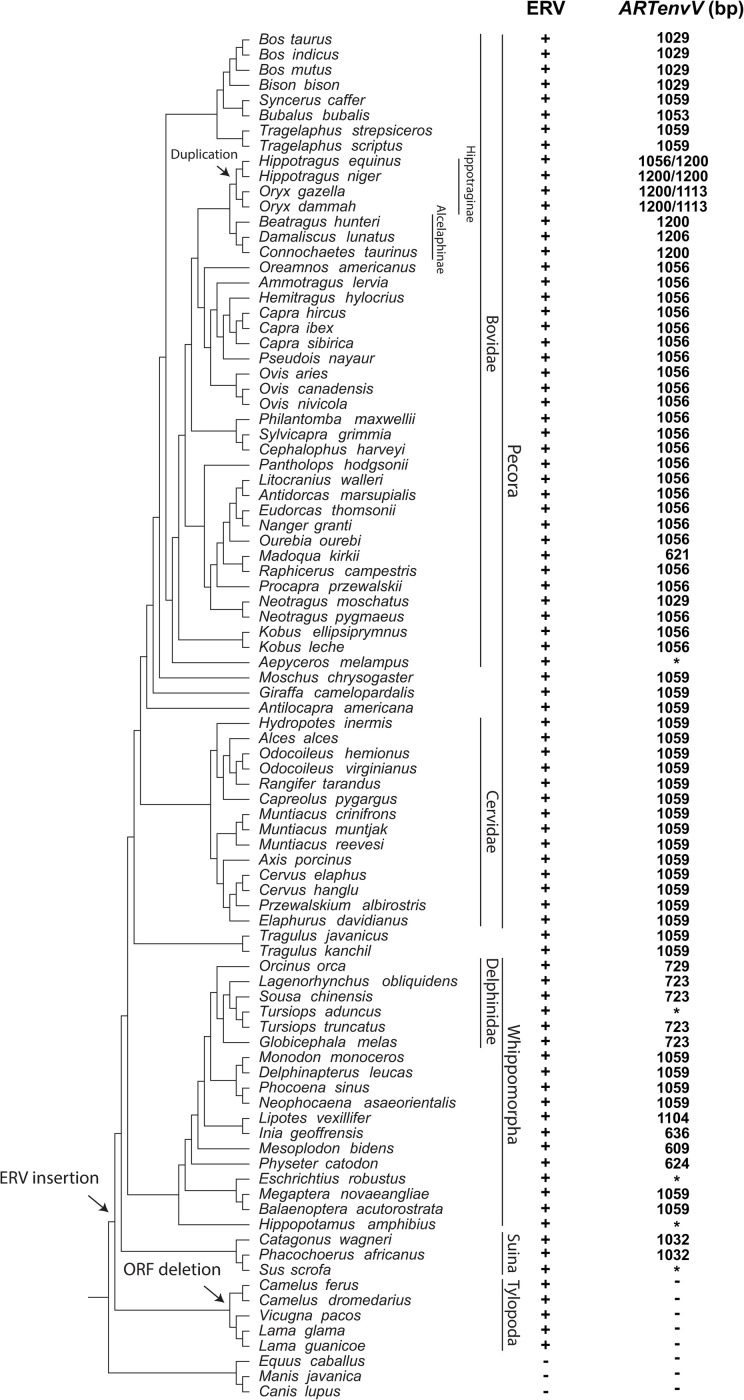
Conservation of *ARTenvV* ORF in the lineages of Artiodactyla. Shown is a cladogram illustrating the evolutionary relationships for species with assembled genomes in the order Artiodactyla along with three species from sister orders of Artiodactyla. Suborder, family and subfamily classifications are shown on the right. The species tree was generated with TimeTree [82]. + indicates the presence and–indicates the absence of ERV orthologs that contain *ARTenvV* as identified via BLAST searches of each genome assembly. The size of the *ARTenvV* ORF found in the orthologous location at each genome assembly is indicated. * indicates a very early stop codon caused by an insertion or deletion. Arrows mark the insertion and fixation of the ERV that encodes *ARTenvV*, retroduplication of *ARTenvV*, and the deletion of the *ARTenvV* ORF.

To determine whether this ERV is found in a region of conserved synteny in other members of Artiodactyla, we extracted and aligned the genomic region surrounding the *ARTenvV* ORF from 17 species of Artiodactyla as well as three other species from the sister orders Perissodactyla (odd-toed ungulates), Pholidota (pangolins) and Carnivora [59,60]. This region is highly conserved in species in the ruminant infraorder Pecora and shows high sequence conservation in the exons of *CREM* confirming the orthology of this genomic region in these orders (Fig 3B). This alignment also shows high conservation of *ARTenvV* in all lineages of Artiodactyla except for the Bactrian camel (*Camelus ferus*) (Fig 3B). The ERV containing *ARTenvV* is also absent in the horse (*Equus caballus*), dog (*Canis lupus familiaris*) and pangolin (*Manis javanica*) genomes (Fig 3B). To describe the evolutionary history of this ERV and confirm the conservation of the *ARTenvV* ORF, we individually inspected this genomic region in all Artiodactyla species that have a genome assembly (S1 Table). Remnants of this ERV are found in all Artiodactyla lineages, and the vast majority of these species also contained >1000 bp of the *ARTenvV* ORF (Fig 4). A few species, including members of the family Delphinidae (oceanic dolphins), contain early stop codons in *ARTenvV* (Fig 4). Notably, all members of the suborder Tylopoda (camels and llamas) have orthologous remnants of this ERV, although none of them contain an *env* ORF as part of this ERV (Fig 4). Detailed comparative analysis of the aligned region containing the *ARTenvV* ERV from *B*. *taurus* and *C*. *dromaderius* (dromedary camel) shows high sequence identity in the first exon of the *CCNY* gene immediately upstream of the *ARTenvV* ERV as well as the regions corresponding to the *gag and pol* portions of this ERV (S1 Fig). However, the Dfam repeat annotation reveals the insertion of a Long Interspersed Nuclear Element-1 (LINE-1) retrotransposon (DF0000255.4) at the location corresponding to the *ARTenvV* ORF in *C*. *dromaderius* (S1 Fig). Hence, these findings indicate that this ERV was fixed in the common ancestor of all extant Artiodactyla which diverged at least 64 million years ago, but that the *ARTenvV* ORF was likely lost in the common ancestor of the extant members of the suborder Tylopoda [60,61].

### Additional copies of *ARTenvV* and *CARenvV* related ERVs

These findings prompted us to determine whether there are other copies of these ERVs in Artiodactyla and Carnivora. Using the ORFs of these genes as probes we identified one additional ERV in both orders with high similarity (~ 70%) to their respective *env* ORFs. While neither of these ERVs contained an intact retroviral gene ORF, analysis of this sequence using the Dfam repeat database indicates that there are remnants of *gag* and *pol* that have the same Dfam designation (Prima41) as the comparable regions of the ERVs that contain *ARTenvV* and *CARenvV* (S2 Fig). To determine whether these ERVs are in a location of conserved synteny, we aligned the genomic region surrounding each ERV from 15 species of Artiodactyla or Carnivora as well as five other species from their sister orders in Laurasiatheria. Each ERV was only found in the same orthologous location in species of their respective orders (S3 Fig). These findings indicate that ERVs derived from a related retrovirus were independently fixed in the common ancestors of Carnivora and Artiodactyla at least 54 and 64 million years ago, respectively [59–61].

### Retroduplication of *ARTenvV*

During the BLASTn search of Artiodactyla using the *B*. *taurus ARTenvV* ORF as a probe, we found an additional copy of this gene in the subfamily Hippotraginae (grazing antelopes). Alignment of the orthologous region of this additional copy showed that it is missing in other Artiodactyla including the Alcelaphinae subfamily which is most closely related to Hippotraginae (S4 Fig). Analysis of the DNA sequence of this copy in a member of this lineage, *Oryx dammah*, shows that it is > 97% identical to the computer annotated mRNA of the *ARTenvV* gene which is predicted to be the spliced product of two exons (S5 Fig). In addition, this extra copy is flanked by 14 bp target site duplications and contains a signature site required for insertion via LINE-1 (TTAAAA) suggesting that this copy represents a LINE-1-mediated retrotransposition. LINE-1 retrotransposons are autonomous non-LTR elements that encode an RNA binding protein and a protein with reverse transcriptase (RTase) and endonuclease function [62]. These elements can use a copy-paste mechanism of reverse transcription (RT) and insertion to recognize and replicate their own mRNAs as well as cellular mRNAs including mRNAs of co-opted ERV derived genes resulting in the insertion of intron-less copies of cellular mRNAs at random genomic locations [62–64]. To confirm the presence of this extra copy of *ARTenvV* we extracted DNA from *O*. *dammah* and two species of Bovidae. Using primer pairs in and around this *ARTenvV* copy, we generated PCR products with the expected size of ~ 700 base pairs (bp) from *O*. *dammah* but not from *B*. *taurus* and *Ovis aries* consistent with the absence of this retrotansposed copy (S6 Fig). Sequencing of these PCR products confirmed this conclusion. Collectively these findings indicate that *ARTenvV* was duplicated via LINE-1 mediated retrotransposition in the common ancestor of the subfamily Hippotraginae at least 7 million years ago [60,61].

### Phylogenetic analysis identifies the *ARTenvV* and *CARenvV* proviruses as gamma-like ERVs

**Fig 5:**
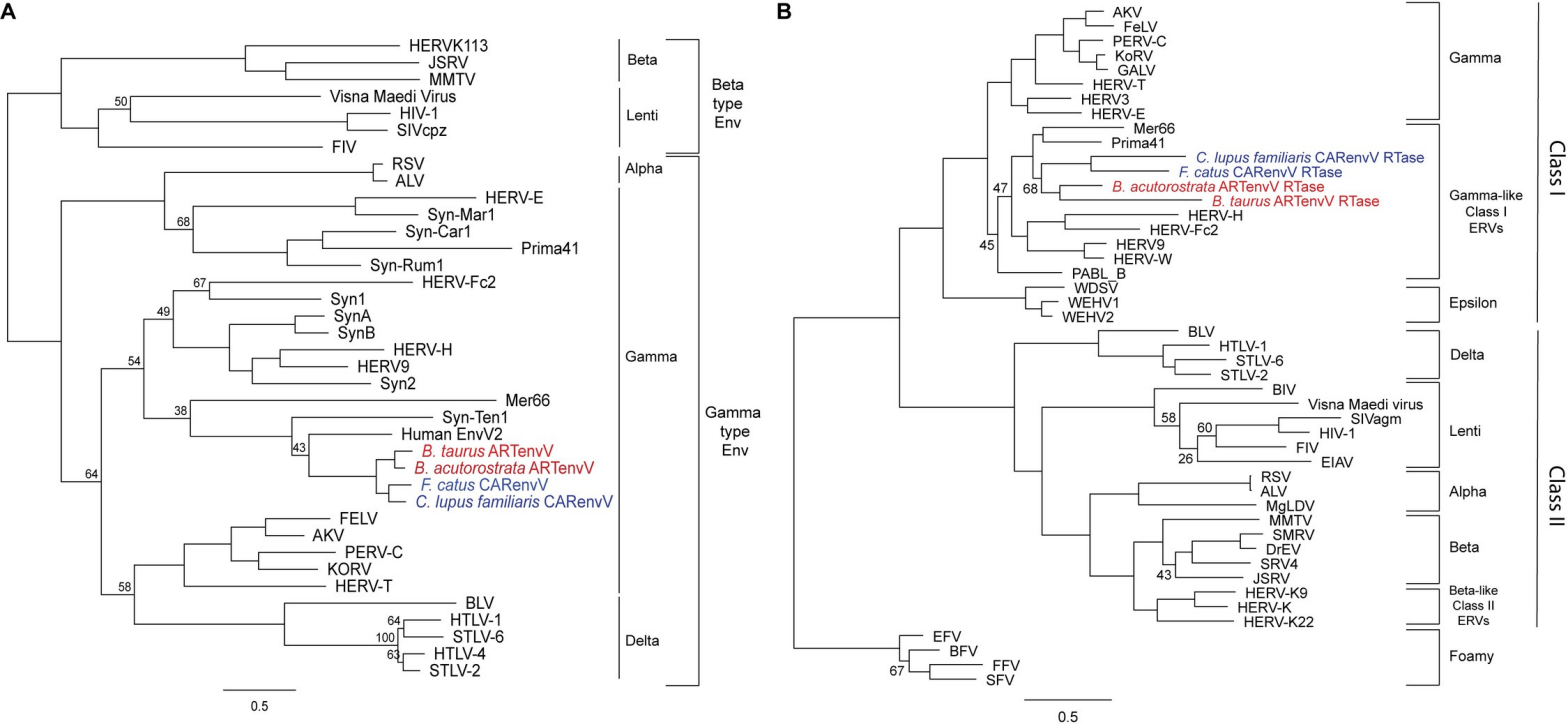
Phylogeny of CARenvV and ARTenvV proteins. **A.** The predicted amino acid sequence of *CARenvV* (in blue) and *ARTenvV* (in red) from the indicated species together with the amino acid sequence of selected endogenous and exogenous retroviral Env proteins (S2 Table) were aligned, and a maximum likelihood tree was generated using RaxML with 500 bootstraps. The tree was midrooted. **B.** Amino acid sequence of the predicted RTase domains of *CARenvV* ERV (in blue) and *ARTenvV* ERV (in red) from the indicated species together with the amino acid sequence of the RTase domain from the indicated endogenous and exogenous retroviruses (S2 Table) were aligned and a maximum likelihood tree was generated using RaxML with 500 bootstraps. The tree was rooted using foamy viruses. Bootstrap values that are less than 70 are shown at the relevant nodes. Scale bar represents 0.5 amino acid substitutions per site.

Retroviruses are classified into two subfamilies; orthoretroviruses and spumaretroviruses [65]. Orthoretroviruses are further subdivided into six genera; alpharetroviruses, betaretroviruses, gammaretroviruses, epsilonretroviruses, lentiviruses, and deltaretroviruses [65]. ERVs are classified based on their similarity to exogenous retroviruses into Class I ERVs (gamma and epsilon), Class II ERVs (alpha, beta, delta and lenti) and Class III ERVs (spuma) [66,67]. While the retroviral classification is based on the RTase domain of the *pol* gene, retroviral envelope proteins are classified into two subtypes, gamma-type and beta-type, based on the nature of the linkage (covalent or non-covalent) between the SU and TM subunits [68]. In a phylogenetic tree of endogenous and exogenous retroviral envelope proteins, CARenvV and ARTenvV cluster with proteins that belong to the gamma-type envelope subgroup with covalently linked SU and TM subunits, a subgroup that includes gamma, delta and alpha-retroviruses (Fig 5A and S2 File) [68]. CARenvV and ARTenvV group with each other and with human EnvV2 (Fig 5A). To identify the retroviral genus that includes the *CARenvV* and *ARTenvV* ERVs, we aligned the amino acid sequence of the *pol* gene RTase domain of exogenous and endogenous retroviruses (S2 Table and S3 File) and generated a phylogenetic tree. As shown in Fig 5B, the identifiable remnants of the RTase of the *CARenvV* and *ARTenvV* ERVs clustered with the exogenous gammaretroviruses and ancient mammalian gamma-like class I ERVs [66,67] together with Prima41 and Mer66 (Fig 5B).

**Fig 6:**
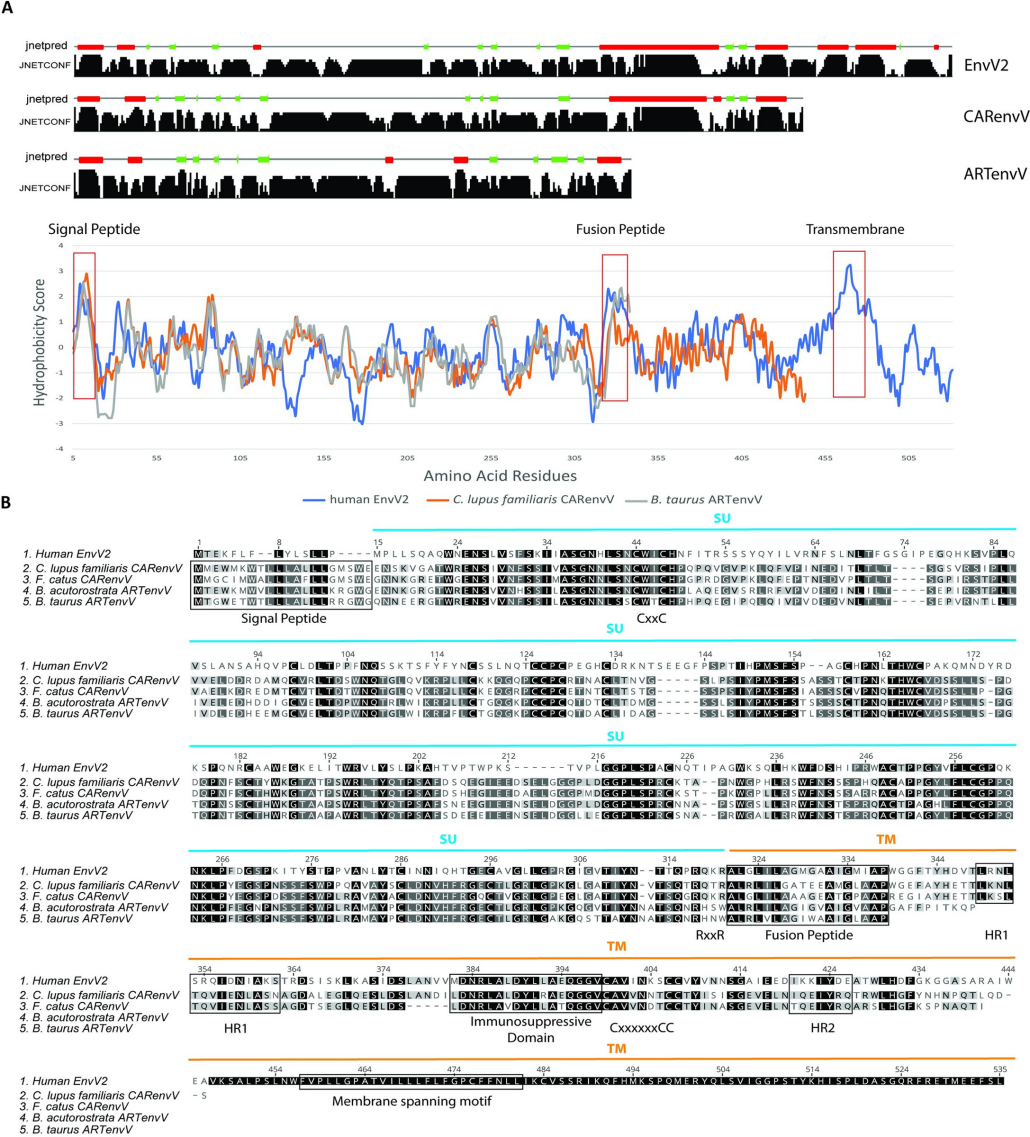
Structural features of CARenvV and ARTenvV. **A.** Secondary structure predictions (top panels) and hydrophobicity profiles (lower graph) are shown for human EnvV2 (blue), *C*. *lupus familiaris* CARenvV (orange) and *B*. *taurus* ARTenvV (gray). Jnetconf shows the confidence levels for the secondary structure prediction while Jnetpred shows the consensus prediction. Red boxes indicate alpha helices and green boxes indicate beta sheets in the secondary structure prediction. The predicted locations of key structural features are indicated on the hydrophobicity profile graph. **B.** Amino acid alignment of human EnvV2, *C*. *lupus familiaris*, and *F*. *catus* CARenvV, and *B*. *taurus* and *B*. *acutorostrata* ARTenvV is shown. Predicted locations of the SU (blue) and TM (orange) domains, signal peptide, the fusion peptide, the immunosuppressive domain, membrane spanning motif, the furin cleavage site (RxxR) between the two domains of the Env, the CxxC and Cx6CC motifs involved in SU–TM interaction are indicated. Predicted heptad repeat regions are indicated as HR1 and HR2. Residues identical in all proteins are indicated by black shading, and residues identical among four, three or two proteins are indicated by different shadings of gray.

To determine whether ARTenvV and CARenvV contain features of retroviral envelope proteins we produced hydrophobicity plots of the ARTenvV and CARenvV proteins together with human EnvV2 and generated the predicted secondary structure of these proteins. Results revealed the presence of a putative N-terminal signal peptide that contains a hydrophobic region and an alpha helix, as well as a predicted fusion peptide in the C-terminal region that also contains a hydrophobic region and an alpha helix (Fig 6A). Amino acid alignment of human EnvV2 together with two orthologs of CARenvV and ARTenvV showed that both these proteins lack a fully intact TMenv, the more highly conserved Env domain. ARTenvV ends after the predicted TMenv fusion peptide and CARenvV is truncated just before the membrane-spanning domain near the *env* C-terminus (Fig 6B). The highest similarity between these Env proteins and human EnvV2 lies in the region immediately following the predicted signal peptide (Fig 6B). This alignment also identified the two conserved cysteine-containing motifs which are found at the same location as in EnvV2: CxxC in the SU domain of both ARTenvV and CARenvV and Cx_6_CC in the TM domain of *CARenvV* (Fig 6B). In addition, the RxxR furin cleavage motif (RQT/KR) is found right before the predicted fusion peptide in the CARenvV but is altered in ARTenvV (Fig 6B). CARenvV also contains an immunosuppressive domain and heptad repeats that show high similarity to the previously identified immunosuppressive domain and heptad repeats of EnvV2 (Fig 6B) [48,49]. The presence of the immunosuppressive domain and the Cx_6_CC motif in the TM domain of CARenvV are consistent with the grouping of this envelope protein with gamma-type envelopes of endogenous and exogenous retroviruses in a phylogenetic tree (Fig 5A) [68]. Taken together, these findings establish that *ARTenvV* and *CARenvV* originated from an ancient gamma-like retrovirus.

### An ancient retroviral cross-species transmission event

Our findings so far suggest that *ARTenvV* and *CARenvV* were independently fixed in the respective common ancestors of extant Artiodactyla and Carnivora. Despite their likely origins from independent infection and endogenization events and their separation by more than 60 million years of evolution, the *ARTenvV* and *CARenvV* ORF orthologs are up to 82% identical (S4 File). In general, the SU domains of retroviral envelopes show high sequence divergence as they must adapt to mutagenized or new receptors and are subject to immune system attack.

**Fig 7:**
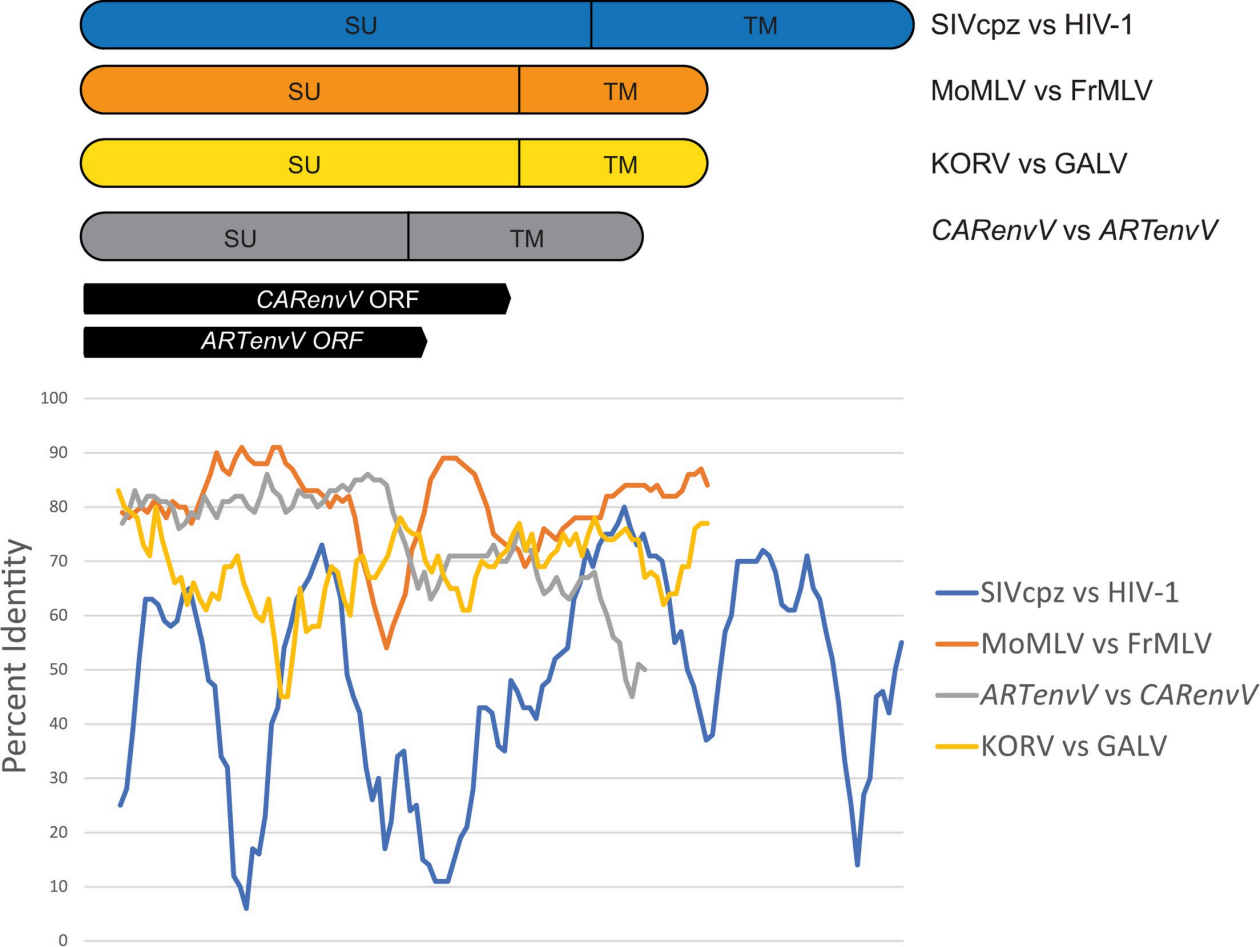
*CARenvV* and *ARTenvV* show high nucleotide identity in SU*env*. Sliding window analysis of percent nucleotide identity along pairwise alignments of *CARenvV* and *ARTenvV* as well as the *env* genes of the indicated retroviruses. The location of the ORFs of *CARenvV* and *ARTenvV* relative to the sequence alignment, the relative sizes of the *env* genes as well as the location of the SU and the TM domains for each alignment are shown above the graph.

To illustrate the extraordinary similarity of *ARTenvV* and *CARenvV* sequences, we aligned the orthologs of the genes with the highest similarity (*Ursus arctos* and *Monodon monoceros*) and performed a sliding window analysis of percent nucleotide identity (Fig 7). Since the ORF of *CARenvV* is longer than *ARTenvV*, and neither of these genes includes the C-terminal end of the TM domain, we included additional nucleotides in this alignment beyond the stop codons of these ORFs using the nucleotide length of *envV2* as a guide (Fig 7). For comparison, we performed the same pairwise analysis for *env* sequences of FrMLV and MoMLV which are derived from the laboratory mouse ecotropic MLV, for KoRV and GALV, which are closely related viruses likely acquired by koalas and gibbons from an intermediate rodent or bat species [24, 25], and for HIV-1 (NC_001802) and its closest relative SIVcpz (AF115393), related by the cross-species transmission of SIVcpz from chimpanzees to humans in the early 20^th^ century [17–19].

Results revealed that the high level of identity in the SU domains of *ARTenvV* and *CARenvV* (80–85%) is comparable to that seen for FrMLV and MoMLV (Fig 7). In contrast, the SU domains of KoRV and GALV show less than 70% identity, while HIV-1 and SIVcpz SUs show only about 60% nucleotide identity and < 70% identity for the entire length of *env* (Fig 7). This demonstrates that *ARTenvV* and *CARenvV* are much more closely related to each other than are the *env* genes of HIV-1 and SIVcpz or KoRV and GALV. These findings suggest that, by the time of their endogenization after cross-species transmission, the progenitors of *ARTenvV* and *CARenvV* had not undergone the same extent of mutational modifications that are observed in the SIV/HIV and GALV/KoRV pairings. Moreover, despite more than 60 million years of evolutionary divergence, *ARTenvV* and *CARenvV* ORFs are still up to 80% identical in nucleotide sequence suggesting that they were much closer in identity prior to their endogenization. Combined with the fact that orthologous copies of each ERV are absent outside of their respective orders, these findings indicate that the original *ARTenvV* and *CARenvV* ERVs likely descended from the same retrovirus that infected both the common ancestor of all the extant members of the order Artiodactyla and the common ancestor of the order Carnivora through an ancient cross-species transmission event.

### Evidence of positive selection in *CARenvV* and *ARTenvV*

The adaptive evolutionary pathways of genes can be traced by comparing the ratio of the rate of nonsynonymous (*dN*) and synonymous (*dS*) changes of their orthologs in related species [69]. Similar to host genes, once endogenized and fixed in a lineage, ERV genes can be conserved under purifying/negative selection to retain a beneficial function leading to a *dN*/*dS* (ω) value below 1, or, they can evolve under diversifying/positive selection, resulting in adaptive changes and producing a ω value above 1 [70,71].

To identify the signatures of selection pressures in *ARTenvV* and *CARenvV*, we separately aligned the ORF orthologs from Artiodactyla and Carnivora. The vast majority of the nodes in the resulting maximum likelihood trees showed high bootstrap support for both *ARTenvV* and *CARenvV* with a few branches with low support in the infraorder Pecora for *ARTenvV* which may be due to its very high sequence similarity in this infraorder (S7 Fig). Additionally, orthologs of both *ARTenvV* and *CARenvV* form clear clusters in the major lineages of each order, including Pecora and Cetacea for *ARTenvV* (S7 Fig) and Caniformia and Feliformia for *CARenvV* (S8 Fig).

Use of the codeml program of PAML4 [72] revealed significant evidence of positive selection for both *ARTenvV* and *CARenvV* (p<0.01) (Table 1). Bayes empirical Bayes (BEB) analysis of posterior probabilities identified six sites for *CARenvV* and two for *ARTenvV* under positive selection with a posterior probability of >0.95 (Table 1) although no sites mapped to homologous sites in the two lineages. Three of these residues for *CARenvV* and one for *ARTenvV* were also found to be under positive selection by the MEME and FEL programs in the Datamonkey webserver [73]. At most sites, however,orthologs of both *CARenvV* and *ARTenvV* showed high sequence similarity, with pairwise ω values much below unity for the vast majority of the species (S5 and S6 Files). These results indicate that while both *ARTenvV* and *CARenvV* are highly conserved, they have each been subject to recurrent positive selection in their respective orders.

**Table 1:** PAML analysis of *CARenvV* and *ARTenvV*.

Gene	Codon frequency	ω^0^^a^	M7-M8		Tree length^c^	*dN/dS* (%)	Residues^d^ with *dN*/*dS* of >1 and pr^e^ of >0.95
			2δ	*P* value			
*CARenvV*	f3 × 4	0.3	9.25	0.0098	2.78555	2.11 (3.45)	56Q, 99Q, 131R*, 336M, 437Y*, 440N*
	f3 × 4	1.7	9.25	0.0098	2.78555	2.11 (3.45)	56Q, 99Q, 131R*, 336M, 437Y*, 440N*
*ARTenvV*	f3 × 4	0.3	11.47	0.0032	2.82655	2.01 (3.61)	240R, 305A*
	f3 × 4	1.7	11.47	0.0032	2.82655	2.01 (3.61)	240R, 305A*

^a^ ω^o^ indicates the initial seed value of ω used.

^b^ 2δ, two times the difference of the natural log values of the maximum likelihood from pairwise comparisons of the different models.

^c^ Tree length is defined as the sum of the nucleotide substitutions per codon at each branch.

^d^ Residue numbers are based on dog (*CARenvV*) and cattle (*ARTenvV*) protein sequences.

^e^ pr, posterior probability.

* Indicates residues identified under positive selection via the datamonkey webserver.

**Fig 8:**
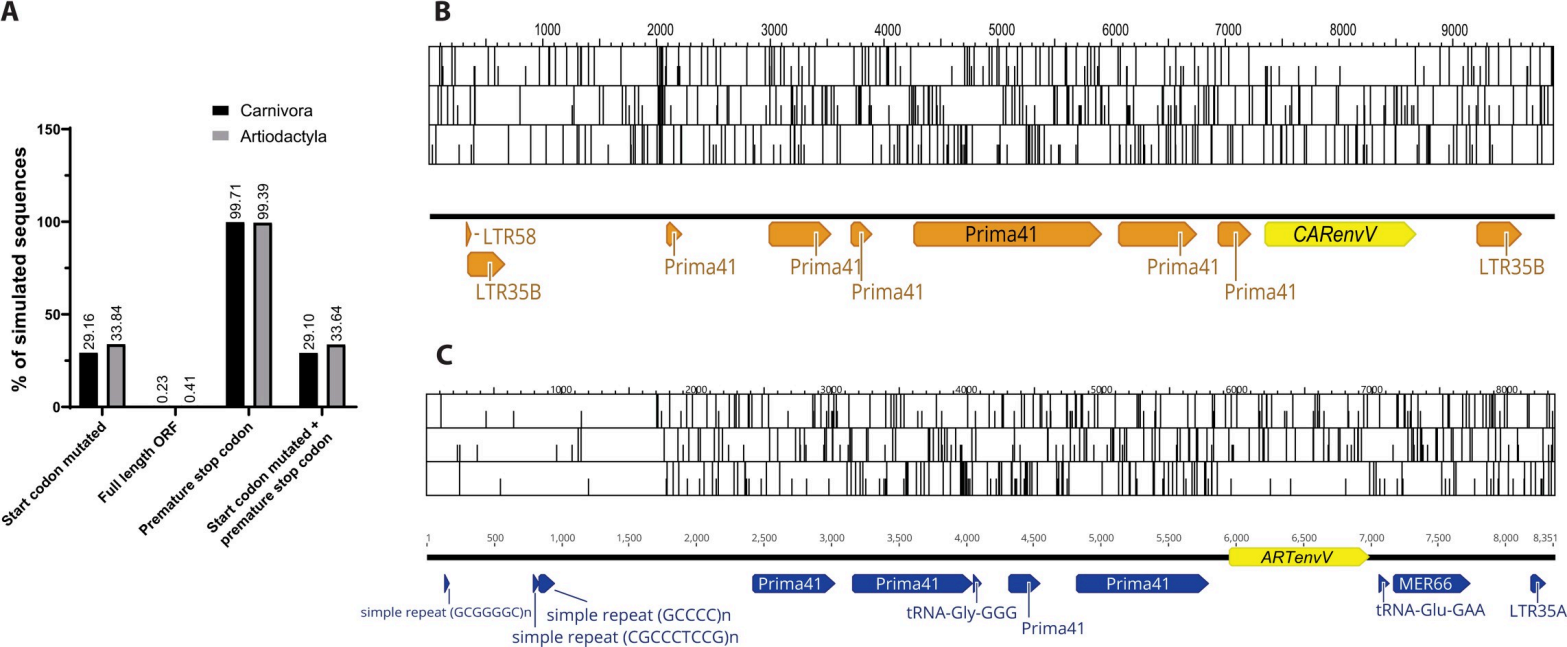
Preservation of *CARenvV* and *ARTenvV* ORF under neutral evolution. **A.** Results of the Monte-Carlo simulations of *CARenvV* and *ARTenvV* evolution for 54 and 64 million years are shown, respectively, using the mammalian neutral substitution rate. The percentage of 100,000 simulated sequences that have the indicated properties are plotted. **B.** ORF map of the *CARenvV* ERV with all three forward reading frames is shown. *CARenvV* ORF is annotated in yellow and the Dfam designation of repeat elements is indicated in orange. **C.** ORF map of the *ARTenvV* ERV with all three reading frames is shown. *ARTenvV* ORF is annotated in yellow and the Dfam designation of repeat elements is indicated in blue. Potential start codons are indicated with a half-height line and stop codons are indicated with a full height line.

Preservation of a large ORF of an ancient retroviral *env* in two mammalian orders for more than 50 million years suggests that evolutionary pressures acted to maintain these ORFs. To determine the level of preservation expected for ORFs this size (1323 bp for *CARenvV* and 1029 bp for *ARTenvV*) under genetic drift and neutral evolution in these orders, we constructed ancestral sequences for both *ARTenvV* and *CARenvV* and simulated their neutral evolution using the mammalian neutral substitution rate [74]. These results revealed that less than 0.5% of the sequences maintained an intact ORF for both *ARTenvV* and *CARenvV*, and the vast majority of the sequences (>99%) contained a premature stop codon (Fig 8A). ORF maps of the ERVs that contain *ARTenvV* and *CARenvV* also reveal the unusual preservation of these *env* ORFs compared to the rest of their proviruses which contain numerous stop codons in all three frames (Fig 8B and 8C). It is important to note that since this analysis does not consider deletions or insertions, we expect the number of intact ORFs to be even lower than what was observed under neutral evolutionary pressure. These findings suggest that selection pressures during the evolution of Carnivora and Artiodactyla led to the preservation and co-option of the *ARTenvV* and *CARenvV* ORFs.

### *CARenvV* and *ARTenvV* are expressed in a variety of tissues

**Fig 9:**
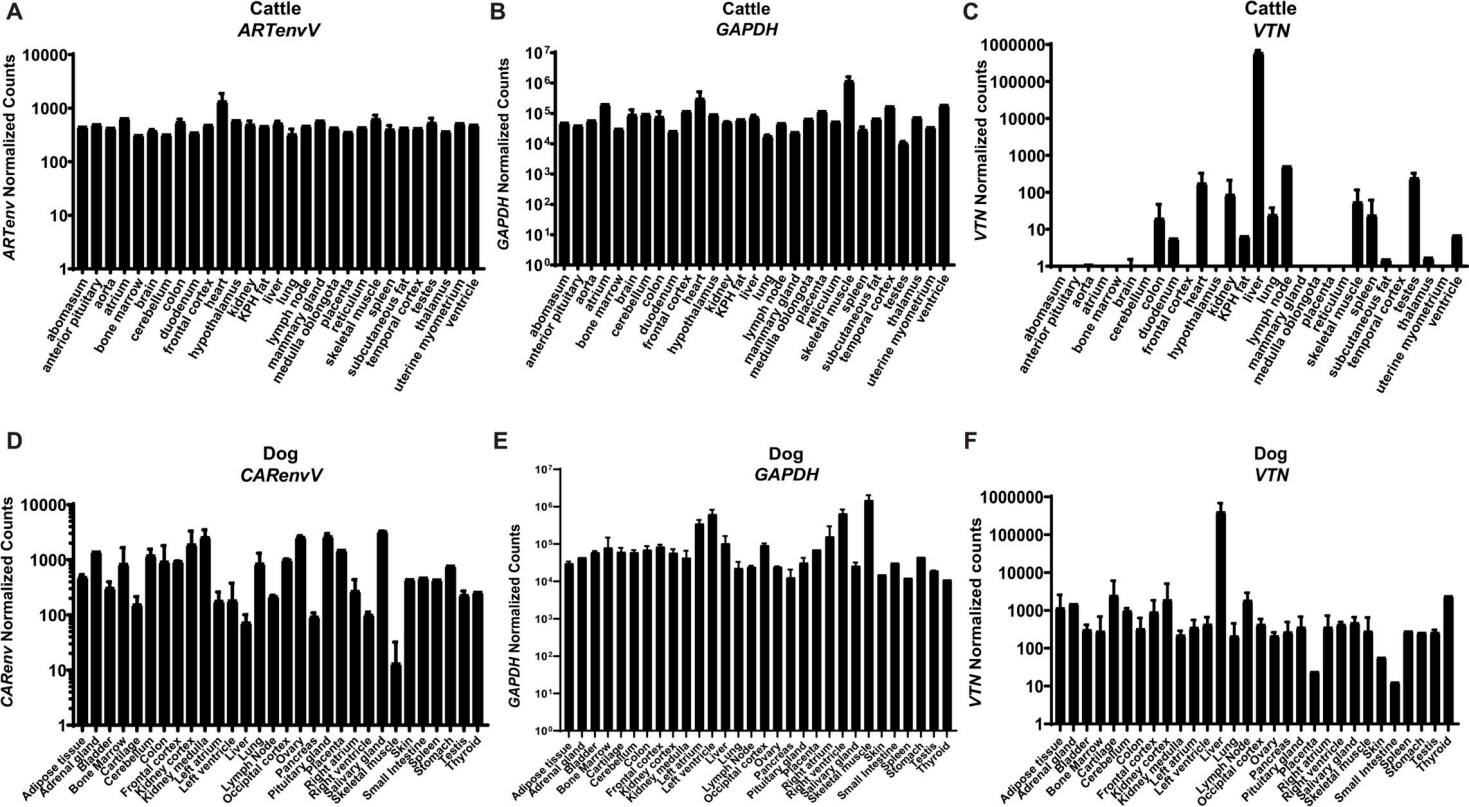
*CARenvV* and *ARTenvV* are expressed in a wide variety of tissues. *In silico* analyses of RNA sequencing data from 29 tissues from cattle (*B*. *taurus*) and 30 tissues from domestic dog (*C*. *lupus familiaris*) are shown for **A.** cattle *ARTenvV*, **B.** cattle *GAPDH*, **C.** cattle *VTN*, **D.** dog *CARenvV*, **E.** dog *GAPDH* and **F.** dog *VTN*. The mapping accuracy of the RNA sequencing reads, and the normalization of the read counts was confirmed using as controls a liver specific gene (VTN) (**C. and F.**) or a housekeeping gene (*GAPDH*) (**B. and E.**). Raw RNA sequencing data extracted from SRA projects PRJNA314981, PRJNA78827, PRJNA379574, PRJNA177791 and PRJNA314981 were mapped to the cattle or dog genome assembly and the counts that map to the coding region of each gene were extracted and normalized using DEseq2. KPH: Kidney, pelvic and heart.

To determine the tissue-specific expression profile of *CARenvV* and *ARTenvV*, we performed *in silico* analyses of RNA sequencing data from the domestic dog (*C*. *lupus familiaris*) for *CARenvV* and from cattle (*B*. *taurus*) for *ARTenvV*. Both *CARenvV* and *ARTenvV* are expressed at similar levels in a wide variety of tissues (Fig 9). While *CARenvV* showed lower expression levels in dog skeletal muscle, liver, pancreas and right ventricle, this was not the case for *ARTenvV* in cattle (Fig 9).

**Fig 10:**
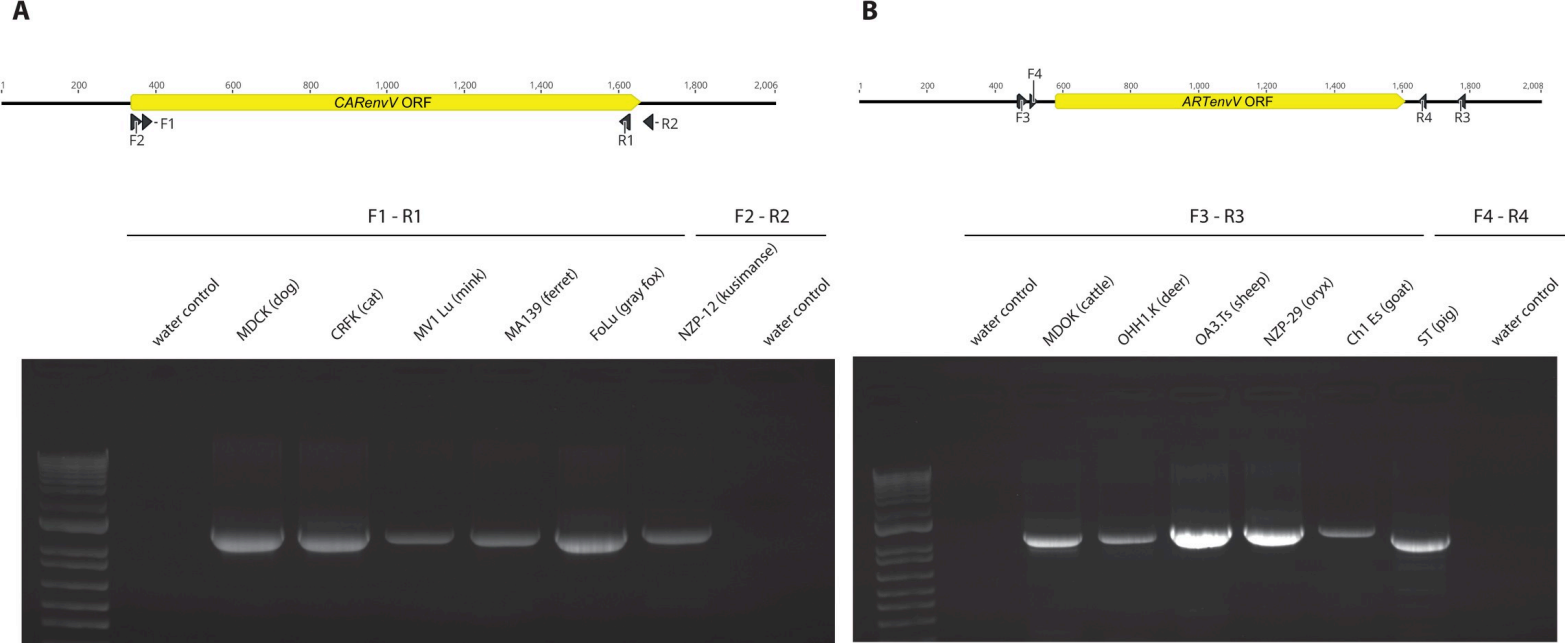
*CARenvV* and *ARTenvV* expression in Artiodactyla and Carnivora cell lines. **A. and B.** At the top is a schematic of the genomic region that contains **A.** *CARenvV* ORF or **B.** *ARTenvV* ORF with the locations of the primers used for RT-PCR indicated below the diagram. Below are the agarose gels showing the RT-PCR products of **A.** *CARenvV* and **B.** *ARTenvV* that were amplified from the total RNA of the indicated species with the primers shown in the upper panel. The gels shown are representative of at least two independent experiments.

Next, we used RT–PCR and 5’ and 3’ RACE-PCR to determine the structure of the transcripts that encode *CARenvV* and *ARTenvV*. First, we probed total RNA extracted from six Carnivora species and six Artiodactyla species for the presence of *CARenvV* and *ARTenvV*, respectively, via RT—PCR. Transcripts were detected in all species tested (Fig 10) and confirmed by sequencing (S7 File). This analysis also showed that both *ARTenvV* and *CARenvV* transcripts are generated via splicing of two exons separated by approximately 4500 and 6500 bp, respectively, and that these genes contain a 3’ UTR ranging from 700 to 1200 bp (S9 and S10 Figs). These findings are also consistent with the computer-based annotation of these genes in RefSeq. For both *ARTenvV* and *CARenvV*, we found the 3’ end of the transcript to be the same among the species analyzed (S9 and S10 Figs), while the transcript start site was within a few nucleotides in different species for ARTenvV (S9 Fig). While 5’ RACE-PCR showed that the transcript start site of *CARenvV* is located within the 5’ LTR of the ERV, we observed significant variation in the location of the transcript start site within the first exon of *CARenvV* among different Carnivora species (S10 Fig). For *ARTenvV*, the transcript start site is located immediately downstream of a GC-rich region (S9 Fig).

### CARenvV and ARTenvV protein expression

**Fig 11:**
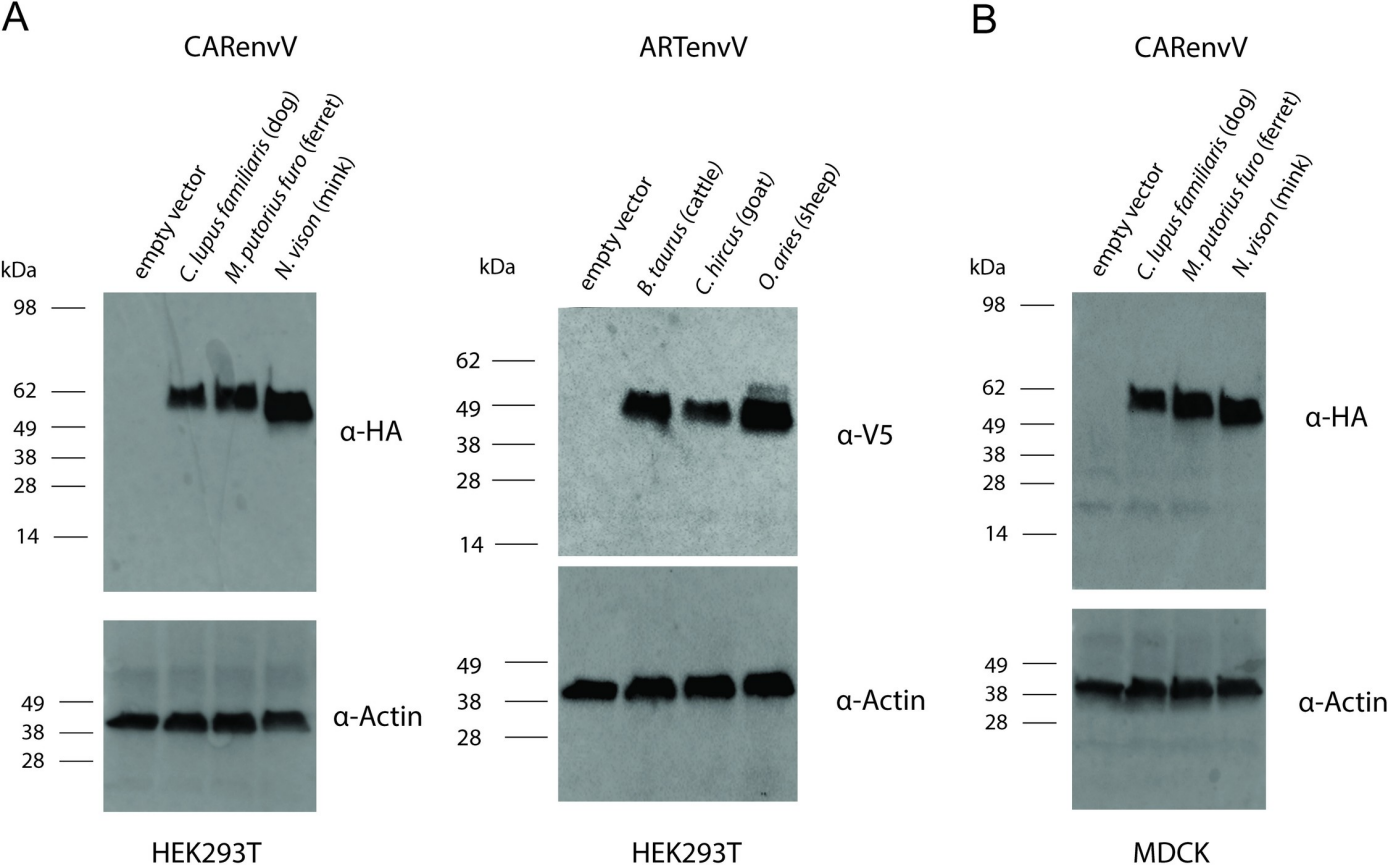
Ectopic expression of CARenvV and ARTenvV. **A.** Immunoblotting analysis of 293T cells transfected with the plasmids expressing empty vector or the indicated *CARenvV* and *ARTenvV* constructs. **B.** Immunoblotting analysis of MDCK cells transduced with the plasmids expressing empty vector or the indicated *CARenvV* constructs. The blots shown are representative of at least two independent experiments.

To determine whether *CARenvV* and *ARTenvV* genes have the capacity to produce envelope proteins, we cloned the ORF of *CARenvV* and *ARTenvV* from three species each into a CMV promoter-driven expression vector, adding C-terminal tags of HA to CARenvV and V5 to ARTenvV. Both CARenvV and ARTenvV proteins from transiently transfected HEK293T cells were detected in whole-cell lysates via immunoblotting (Fig 11A). Both ARTenvV and CARenvV contain multiple potential N-linked glycosylation sites, and their observed molecular weights are higher than the expected weights of 37.6 and 49.1, respectively, suggesting a glycosylation mediated shift. In addition, while the amino acid sequence of CARenvV (but not ARTenvV) contains a predicted furin cleavage site (Fig 6B), we did not observe any evidence of cleavage of CARenvV protein when expressed in HEK293T cells (Fig 11A). To determine whether CARenvV is subject to furin cleavage in cells from a Carnivora species, we expressed HA-tagged CARenvV from three species in MDCK cells using a retroviral delivery system. As shown in Fig 11B, we observed expression of CARenvV at the sizes expected for uncleaved proteins but observed no smaller cleavage products. These findings show that both *CARenvV* and *ARTenvV* ORFs can produce stable proteins under the control of a constitutive promoter when exogenously expressed.

## Discussion

**Fig 12:**
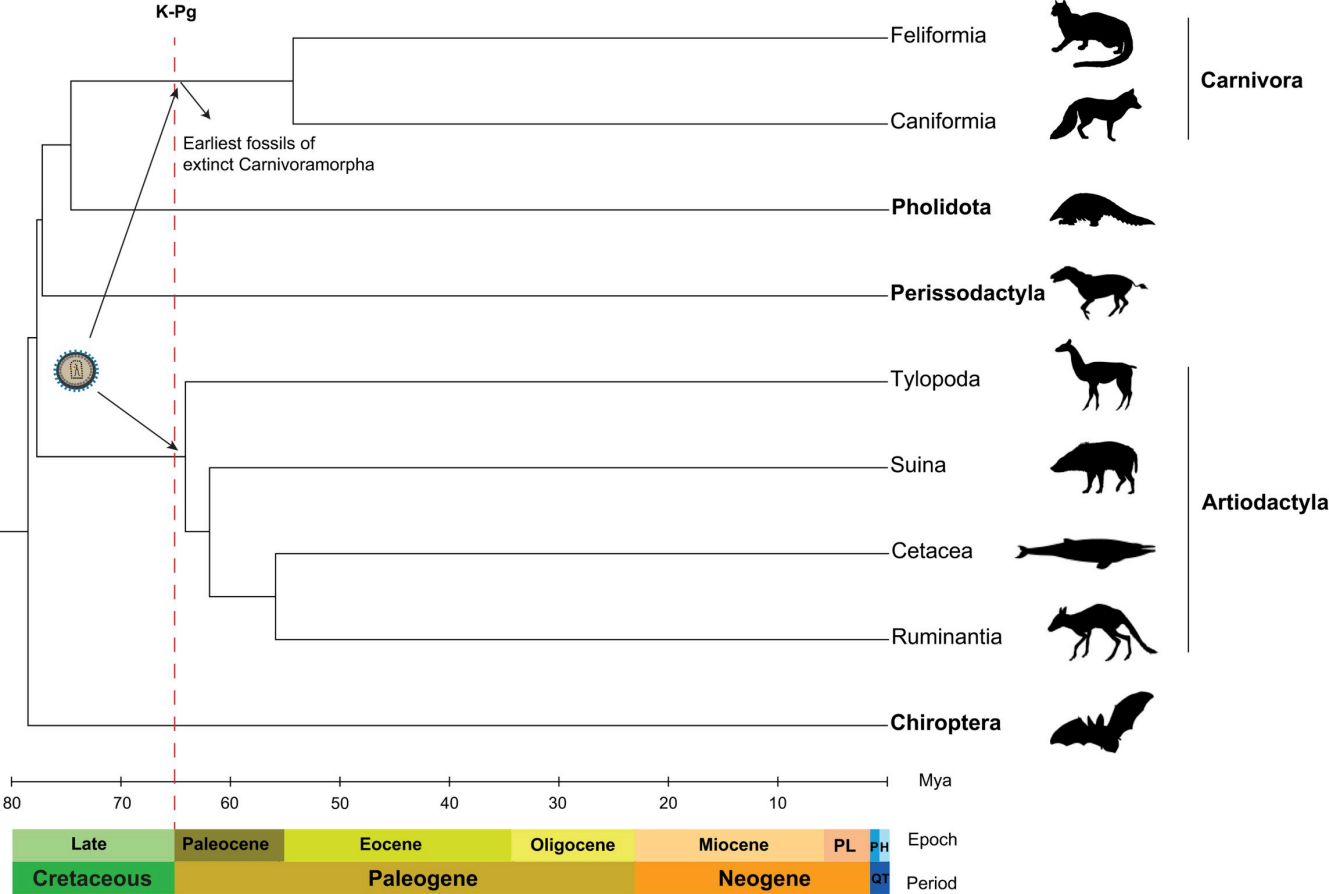
Cross-species transmission of an ancient gammaretrovirus around the K-Pg event. The species cladogram shows the phylogenetic relationships for the indicated orders (in bold) that belong to the superorder Laurasiatheria. Suborders are identified for Carnivora and Artiodactyla. The tree and the divergence times were generated using TimeTree [82]. Geological timescales were derived from The International Chronostratigraphic Chart [119]. PL; pliocene, QT; quaternary, P; pleistocene, H; holocene.

The Cretaceous–Paleogene (K–Pg) mass extinction event that is thought to have killed the non-avian dinosaurs approximately 66 million years ago represents a major turning point in the evolutionary history of Eutheria (placental mammals) [75]. While there are multiple models of the impact of the K-Pg event on the interordinal diversification of Eutheria [76], both the fossil record and molecular clock studies suggest that placental mammalian orders went through rapid diversification following the K-Pg event that continued throughout the Paleocene and Eocene epochs leading to the emergence of the modern taxa [77,78]. In this study, we presented multiple lines of evidence that a gamma-like retrovirus crossed the species barrier and was independently fixed in the genomes of the ancestral species of Artiodactyla and Carnivora during this significant period in the history of placental mammals (Fig 12). First, we found no evidence of *ARTenvV* and *CARenvV* orthologs in species outside of their respective orders including the Artiodactyla sister orders Perissodactyla and Pholidota. An alternative scenario based on a single endogenization event and vertical inheritance from the common ancestor of all these mammalian orders would have required three independent deletion events involving different ERVs in the common ancestors of three different mammalian orders. A much more likely scenario is that these ERVs were horizontally and independently acquired by the common ancestors of all extant Artiodactyla and Carnivora. This hypothesis is also bolstered by the fact that we found a highly similar ERV in separate orthologous locations in both Artiodactyla and Carnivora (S3 Fig) while other mammalian taxa lack this ERV subtype.

Additionally, the preserved *env* ORFs of this ancient ERV (*ARTenvV* and *CARenvV*) found in the extant members of Artiodactyla and Carnivora display an unusually high sequence similarity (75–82%) in the ordinarily divergent SU*env* domain that is much higher than the nucleotide identity observed between other pairs of closely related retroviruses that are formed as a result of a cross-species transmission event (e.g. HIV-1 and SIVcpz) [17–19]. It is not feasible for two ERVs that were not derived from the same virus to have such high nucleotide similarity in the otherwise highly variable SU domain of the *env* gene, indicating that *ARTenvV* and *CARenvV* were most likely formed as a result of an ancient cross-species transmission event involving the same gamma-like retrovirus.

Estimates from molecular clock studies using fossil calibrations indicate that the modern Carnivora diverged from their most recent common ancestor in the early Eocene epoch approximately 54 million years ago (Fig 12) [59,60]. However, paleontological studies group the extant Carnivora lineages with ancient extinct carnivorans into a stem-group called Carnivoramorpha based on phenotypic characteristics identified in the fossils [79]. This stem-group includes members that are thought to have lived during the Paleocene epoch (66 to 55 mya) based on the fossil record [80, 81]. In addition, molecular clock studies using both nuclear and mitochondrial genes put the divergence of the Artiodactyla from a common ancestor around the K-Pg event, in the early Paleocene or late Cretaceous between 67 and 61 mya [55,60,61,82]. Hence, it is conceivable that the exogenous retrovirus that produced *ARTenvV* and *CARenvV* transmitted directly between an ancestral Carnivoramorpha and Artiodactyla (Fig 12). Modern carnivorans are known to prey on even-toed ungulates, especially ruminants. The oldest known Artiodactyla fossil belongs to an extinct genus called *Diacodexis* [79,83], members of which resemble the modern mouse-deer (family Tragulidae), a small ruminant and a prey species of felines in Asia [84]. Although the remnants of *Diacodexis* do not appear in the fossil record until the Eocene epoch [83], it is feasible that ancestors of this genus that lived alongside the ancestors of Carnivoramorpha around the K-Pg event had a prey-predator relationship. Predation is a major mode of viral transmission between different mammalian species [85]. In fact, the major mode of transmission of SIVcpz to humans leading to the formation of HIV-1 is thought to be bushmeat hunting in western Africa which likely exposed hunters to the bodily fluids of SIVcpz-infected chimpanzees [86,87].

The possibility remains that cross-species transmission of this retrovirus happened after it was endogenized and fixed in one lineage. Some ERVs stay active even after being fixed in a population [28,88,89]. It is feasible that the exogenous gamma-like retrovirus that gave rise to *ARTenvV* became endogenized in the common ancestor of the Artiodactyla in the early Paleocene epoch and stayed replication-competent until the Eocene at which time the exogenous version of this ERV infected the germline of the common ancestor of the extant Carnivora. However, because we only found one extra copy of this ERV in both Artiodactyla and Carnivora, even if it stayed active for an extended period, it did not amplify or leave a recognizable fossil record in extant species. It is also possible that there were intermediate species involved in the transmission of this ancient virus. GALV and KORV are highly related gammaretroviruses hypothesized to have been acquired from an intermediate bat or rodent species [24,25,90]. Retroviral endogenization and fixation in a lineage is a rare event. Hence an intermediate species could have been infected with the progenitor gamma-like retrovirus of *ARTenvV* and *CARenvV* during the Paleocene epoch, and either this virus was not endogenized in this lineage or all of its members became extinct.

It is highly likely that evolutionary selection pressures kept the SU domain of *ARTenvV* and *CARenvV* intact, maintaining their unusual sequence similarity over more than 60 million years of evolution. Our sequence simulation analysis suggests that an ORF that is >1000 bp would acquire stop codons more than 99.5% of the time under neutral evolution in this 55–65 million year timeframe. However, the large ORFs in both *ARTenvV* and *CARenvV* are conserved with high sequence similarity in all lineages of their respective orders with only a few species acquiring early stop codons (Figs 2 and 4). Although we identified signatures of positive selection in both *ARTenvV* and *CARenvV*, *dN*/*dS* values were much below unity for the vast majority of the species. A few positively selected residues identified in CARenvV and ARTenvV were concentrated at the SU domain (3/6 for CARenvV and 2/2 for ARTenvV) even though none of them were located near each other. These residues may represent functional adaptations to the yet unidentified receptor of these *env* genes. *env* genes of circulating retroviruses tend to be under strong positive selection as they are in an arms race with the immune system of the host [91]. Our findings of limited positively selected residues for ARTenvV and CARenvV are consistent with these proteins being selected and conserved as host genes and are in concert with other co-opted *env* genes identified in mammals [34,37–42,48,92]. Future studies on the function of these *env* genes may reveal the functional importance of the positively selected residues we identified.

Our findings also offer additional clues to the origins of *CARenvV* and *ARTenvV*. Firstly, protein sequences of *CARenvV* and *ARTenvV* show similarity to human EnvV2 (Fig 6B), a co-opted ERV derived *env* gene found in simian primates [48,49] suggesting that these ERVs may share a common ancestor. In addition, repeat annotation of *CARenvV* and *ARTenvV* ERVs revealed that different portions of these sequences show similarity to two different primate ERV subfamilies, Prima41 and MER66 (Figs 1 and 3). This finding indicates that these ERVs may have experienced recombination. However, since both *CARenvV* and *ARTenvV* ERVs contain Prima41-like *gagpol* and Mer66-like *env* sequences, any potential recombination likely happened before endogenization and cross-species transmission.

While our results indicate that both *ARTenvV* and *CARenvV* are expressed in a variety of tissues, and the ORFs of these genes are capable of producing stable proteins when expressed from a constitutive promoter, the reason for the preservation of these retroviral genes for more than 60 million years is not clear. Most of the previously identified co-opted retroviral *env* genes contain TM domains and are fusogenic [31–38,40,42,48]. However, the transmembrane spanning portion of *env* is lost in both *CARenvV* and *ARTenvV* in all lineages. Hence, it is likely that both these genes acquired premature stop codons early in the evolution of each order before the divergence of the modern lineages and that the fusogenic function of these *env* genes either did not contribute to or conflicted with the basis for their co-option. Previous studies identified non-fusogenic/secreted co-opted *env* genes in mammalian genomes. For example, suppressyn, a truncated ERV derived Env protein found in simian primate genomes, is a soluble inhibitor of syncytin-1 induced cell fusion in the placenta [46]. While the widespread tissue expression profiles of *CARenvV* and *ARTenvV* make it unlikely that these genes are co-opted for a placenta specific function, we cannot rule out a function related to interference with circulating retroviruses that bind the same receptor, similar to some ERV envelope derived genes identified in mice and cats [93–95]. These questions may be addressed by the resolution of the tertiary structures of CARenvV, ARTenvV and EnvV2, and by the identification of the cell surface receptors for each protein. It is also important to note that while we detected the expression of *CARenvV* and *ARTenvV* ORFs by exogenously expressing the C-terminally tagged versions of these proteins, further studies using custom antibodies against these proteins in primary cells from relevant species could determine their endogenous expression levels and subcellular locations. Future studies may also determine the ancestral tropism/receptor of these envelope proteins by reconstructing the mutated TM region and testing fusion function in different cell types [96].

Our findings also revealed the presence of a retroduplicated, intronless copy of the *ARTenvV* gene in the members of the Hippotraginae, a subfamily in the order Artiodactyla. This copy of *ARTenvV* contains hallmarks of a LINE-1 mediated retrotransposition; an intronless copy of a gene at a different genomic location, a target site duplication at each side and a short series of As immediately downstream of the duplicated copy. LINE-1 mediated retrocopying of genes is a significant source of genomic diversity in mammals. Computational surveys done in the last two decades identified thousands of retroduplicated copies of genes including ERVs in the genomes of various mammals [97–100]. While the majority of these retrocopies are pseudogenes that have lost coding capacity, several retrogenes have been shown to express proteins, some of which have evolved new functions or become part of novel fusion genes by merging with other transcribed sequences [101–105]. Due to the extremely high sequence similarity (>97%) between the original and the retroduplicated copy in the *O*. *dammah* genome, we were not able to determine whether the retroduplicated copy is expressed in this species. However, all species belonging to the Hippotraginae with a genome in the database maintained an intact ORF of the retroduplicated *ARTenvV*. This may suggest that either this extra copy is still expressed in these species due to evolutionary pressures that maintain the ORF or simply that not enough time has passed since the retroduplication for the accumulation of enough mutations to neutralize this copy. Further analysis of RNAseq data from multiple tissues of *O*. *dammah* or other Hippotraginae species may determine if this intronless retroduplicated copy of *ARTenvV* is still expressed. We believe this is the first discovery of a retroduplicated copy of a co-opted retroviral *env* gene.

The oldest previously reported cross-species transmission events of retroviruses among mammals were proposed to have happened in the Oligocene epoch (24–34 million years ago) [8,28]. Our findings indicate a much older cross-species transmission of an ancient gamma-like retrovirus early in the age of mammals (Cenozoic Era) at least 64 million years ago, before the divergence of the extant lineages of Carnivora and Artiodactyla. These findings highlight the complex evolutionary relationship between retroviruses and the placental mammals as *ARTenvV* and *CARenvV* represent the first discovery of convergent co-option of the envelope gene from the same parental retrovirus in different mammalian orders.

## Methods

### Search for HERV-V related ERVs in mammalian genomes

The amino acid sequence of human *envV2* (NP_001177984.1) was used as a probe for the tBLASTn search of the annotated mammalian genomes in the NCBI database (https://www.ncbi.nlm.nih.gov/genome/annotation_euk/all/) using the web based BLAST program available at the NCBI website (https://blast.ncbi.nlm.nih.gov). tBLASTn utilizes an amino acid sequence as a query and searches for all six frames of a given DNA sequence. The following parameters were used for tBLASTn: gap costs, 11 and 1 (existence and extension); matrix, BLOSUM62; conditional compositional score matrix adjustment; Expect threshold, 10^−100^. The DNA sequence of the hit from the initial tBLASTn search found in the genome of *Ursus maritimus* was extracted using the NCBI genome viewer (https://www.ncbi.nlm.nih.gov/genome/gdv/). This sequence was used in a subsequent BLAST search of the whole genome shotgun contigs of all Carnivora genomes housed in the NCBI genome database. The following parameters were used for BLASTn: gap costs, 5 and 2 (existence and extension); match/mismatch scores, +2/−3; repeat masking filter turned off; Expect threshold, 10^−100^.

To identify other mammalian ERVs that show similarity to *CARenvV*, the ORF of *C*. *lupus familiaris CARenvV* was used in a BLASTn search of annotated mammalian genomes excluding Carnivora. This search used the same parameters for BLASTn as above.

### Reagents and plasmids

293T, MDBK (*B*. *taurus*), OHH1.K (*O*. *hemionus*), OA3.Ts (*O*. *aries*), Ch1 Es (*C*. *hircus*), ST (*S*. *scrofa*), NZP-29 (*O*. *dammah*), MDCK (*C*. *lupus familiaris*), CRFK (*F*. *catus*), Mv1 Lu (*N*. *vison*), FoLu (*U*. *cineroargenteus*) and NZP-12 (*C*. *obscurus*) cells were obtained from ATCC (Manassas, VA). Ferret (*M*. *putorius furo*) MA139 cells were obtained from Dr. Janet Hartley (NIAID, Bethesda, MD). Each cell line was grown in DMEM (Thermo Fisher, Waltham, MA) supplemented with 1% penicillin and 100 μg/mL streptomycin and 10% fetal bovine serum.

The PCR 2.1 TOPO and pcDNA-V5-TOPO plasmids were purchased from Thermo Fisher. The pLNCX2 and pVSV-G plasmids were obtained from Clontech (Mountain View, CA). The pCMV*gagpol* plasmid which encodes the *gagpol* gene of the Moloney murine leukemia virus was obtained from Cell Biolabs (San Diego, CA). The pcDNA 3.1 plasmid with a C-terminal HA tag was a kind gift from Dr. Klaus Strebel (NIAID, Bethesda, MD).

Anti-HA and anti-V5 mouse monoclonal antibodies were obtained from Thermo Fisher. Anti-Beta-actin rabbit polyclonal antibody was obtained from Sigma-Aldrich (St. Louis, MO). Goat anti-rabbit-HRP and rabbit anti-mouse-HRP antibodies were purchased from Southern Biotech (Birmingham, AL). G418 sulfate was obtained from Corning (New York, NY).

### Database search for *CARenvV* and *ARTenvV* orthologs

*CARenvV* and *ARTenvV* ORF sequences from the *C*. *lupus familiaris* and *B*. *taurus* genome assemblies were used as probes in a BLAST search of 66 Carnivora and 125 Artiodactyla genome assemblies housed in the Genbank database. The following parameters were used for BLASTn: gap costs, 5 and 2 (existence and extension); match/mismatch scores, +2/−3; repeat masking filter turned off; Expect threshold, 10^−75^. 51 species of Carnivora that contained the *BTN1A1* and *BTN2A1* genes and 87 species of Artiodactyla that contained at least 25 kb long sequence surrounding the *ARTenvV* in a single scaffold/chromosome were used for further analysis (S1 Table).

### DNA and RNA isolation

Total RNA was isolated from MDBK, OHH1.K, OA3.Ts, Ch1 Es, ST, NZP-29, MDCK, CRFK, MA139, Mv1 Lu, FoLu and NZP-12 cells using the PureLink RNA Mini Kit (Thermo Fisher), following the manufacturer’s instructions. Genomic DNA was isolated from OA3.Ts, MDBK and NZP-29 cells using PureLink Genomic DNA Mini Kit (Thermo Fisher), according to the manufacturer’s instructions.

### 5’ and 3’ RACE, RT-PCR, PCR and sequencing

RT—PCR for total RNA samples was performed using the Superscript III one step RT-PCR kit (Thermo Fisher) with the following program: 55°C for 30min; 95°C for 3min; 35 cycles of 95°C for 30s, 58°C for 30s, and 72°C for 120s; and 72°C for 5min. 5’ and 3’ RACE-PCR on total RNA isolated from cell lines was performed using SMARTer RACE 5’/3’ kit (Clontech) following manufacturer’s instructions. Primers used in this study are provided in S3 Table.

PCRs for amplifying the region surrounding the retrotransposed copy of *ARTenvV* ORF in genomic DNA samples were performed using Amplitaq Gold (Thermo Fisher) with the following program: 95°C for 3 min; 35 cycles of 95°C for 30 s, 60°C for 30 s, and 72°C for 90s; and 72°C for 5min using the relevant primers (S3 Table).

RT—PCR and PCR products were analyzed by 1% agarose gel electrophoresis and cloned into the PCR 2.1 TOPO plasmid (Thermo Fisher), following manufacturer’s instructions before sequencing. Gel images were captured using GENE FLASH (Syngene, Frederick, MD).

### Plasmid construction for cDNA expression

For the construction of the plasmids that express C-terminally HA-tagged *CARenvV* and V5-tagged *ARTenvV*, total RNA from MDCK, Ch1 Es, OA3.Ts, Mv1 Lu, MDBK and MA139 cells were subjected to RT-PCR Superscript III one step RT-PCR kit (Thermo Fisher) with the following program: 55°C for 30 min; 95°C for 3 min; 35 cycles of 95°C for 30 s, 58°C for 30 s, and 72°C for 120 s; and 72°C for 5 min using the relevant primers indicated in S3 Table. PCR products were gel extracted using QIAquick gel extraction kit (QIAGEN, Germantown, MD). *ARTenvV* ORFs were cloned into pcDNA–V5 –TOPO plasmid with the TOPO cloning strategy using the manufacturer’s instructions and *CARenvV* ORFs were cloned into pcDNA3.1 plasmid with a C-terminal HA tag using EcoRI and EcoRV restriction enzymes.

### Immunoblotting and detection of Env protein

293T cells were grown in a 12 well plate with 200,000 cells/well and transfected with 1000 ng of either of the following plasmids: pcDNA–*ARTenvV(C*. *hircus)*–V5, pcDNA–*ARTenvV(O*. *aries*)–V5, pcDNA–*ARTenvV*(*B*. *taurus*)–V5, pcDNA–*CARenvV(C*. *lupus familiaris)*–HA, pcDNA–*CARenvV*(*N*. *vison*)–HA, pcDNA–*CARenvV(M*. *putorius furo)*–HA and pcDNA–empty. Each transfection was done using Fugene HD (Promega). Three days after transfection, equal numbers of cells were lysed using RIPA buffer (Thermo Fisher) and lysates were sonicated with a Q700 sonicator (QSonica, Newtown, CT). For SDS-PAGE, equal volumes of lysates were loaded onto a 4–12% Bis Tris polyacrylamide gel, and immunoblotting was performed on a PVDF membrane after the transfer. Images of the immunoblots were produced using the ChemiDoc MP imaging system (Bio-Rad, Hercules, CA).

### Retroviral transduction

For retrovirus mediated transduction of *CARenvV* into MDCK cells, *CARenvV* ORF sequences with an HA tag from the following species/plasmids were subcloned into the pLNCX2 retroviral plasmid using XhoI and HindIII restriction enzymes: pcDNA-*CARenvV (C*. *lupus familiaris)*–HA, pcDNA–*CARenvV*(*N*. *vison)*–HA, pcDNA–*CARenvV (M*. *putorius furo)*–HA.

For retroviral vector production, HEK293T cells were seeded at 70% confluency in 12 well plates and transfected with 500 ng of either of the following plasmids; pLNCX2–*CARenvV* *(C*. *lupus familiaris)*–HA, pLNCX2–*CARenvV*(*N*. *vison)*–HA, pLNCX2–*CARenvV(M*. *putorius furo)*–HA, pLNCX-empty and 400 ng pCMV*gagpol* and 100 ng pVSVG plasmids using Fugene HD. Three days post-transfection, the supernatant was collected and filtered through 0.45 μM filters (Millipore, Burlington, MA). 500 uL of the filtered supernatant was used to infect MDCK cells plated into a 6 well plate using 100000 cells/well. Three days post-transduction, 500 ug/mL G418 was added to the media and the transduced cells were grown under selection for three weeks before immunoblotting analysis was performed as described above.

### Sequence alignment and phylogenetics

*CARenvV* and *ARTenvV* ORF sequences were aligned using MUSCLE as implemented in Geneious Prime using default settings. For pairwise analysis of aligned *CARenvV*-*ARTenvV*, FrMLV-MoMLV, KORV-GALV and HIV-1-SIVcpz *env* genes, SimPlot software was used with a 120 bp window size and step size of 20 bp [106]. Maximum-likelihood phylogenetic trees were generated using the RaxML program with the General Time Reversible + G + I model and 500 bootstraps for branch support [107].

For the analysis of the region of conserved synteny among Laurasiatheria for *ARTenvV*, *CARenvV* as well as additional copies of related ERVs, we examined the genomic region that encompasses *BTN1A1* and *BTN2A1* (*CARenvV*) as well as regions encompassing 150 kb downstream of *SPTLC3* (the second Carnivora ERV copy), 50 kb downstream of *CREM* (*ARTenvV*) and 50 kb upstream of *LDLRP1B* (the second Artiodactyla ERV copy). These segments were extracted from the most recent Genbank assembly of each species (S1 Table). Multipipmaker was used for sequence alignment [108].

To determine the location and the sequence of the *pol* region for the RTase domain in the ERVs that contain *CARenvV* or *ARTenvV*, we extracted the genomic sequence 5000 bp upstream of the start codon of the *CARenvV* or *ARTenvV* ORF from four species (*F*. *catus*, *C*. *lupus familiaris*, *B*. *taurus* and *B*. *acutorostrata*). We queried these sequences via tBLASTn using the RTase domain of the Prima41 Pol protein sequence. The location of the RTase domain in the curated Prima41 Pol protein sequence (Dfam accession number: DF0001052.4) was identified by searching the Pfam [109] and Supfam [110] databases. The following parameters were used for the tBLASTn search: gap costs, 11 and 1 (existence and extension); matrix, BLOSUM62; conditional compositional score matrix adjustment; expect threshold, 10^−20^. Due to frameshifts accumulated over the evolution of these ERVs, the amino acid sequence from these searches was in different frames for some species. For the final alignment used in tree construction, amino acid sequences in different frames were juxtaposed to form the final sequence used for analysis. This strategy was also used to identify the RTase domain sequences of the ERV derived *pol* genes used for phylogenetic tree construction. RTase domain sequences of retrovirus sequences found in Genbank were identified either using the annotated sequence (where available) or by using the Pol protein sequence as a query for searches of the Pfam and Supfam databases. Amino acid alignments used for tree construction are provided in S2 (Env) and S3 (RTase) Files.

### Secondary structure prediction for ARTenvV and CARenvV

Amino acid sequences of *C*. *lupus familiaris* CARenvV, *B*. *taurus* ARTenvV and human EnvV2 were used as queries for the Jpred secondary structure prediction server [111]. Jnetpred and Jnetconf graphs are extracted and used for determining secondary structure (Fig 6A). Hydropathicity scores for the same proteins were plotted using ProtScale (https://web.expasy.org/protscale/).

### LTR dating estimation analysis

To estimate the date of integration for the ERV containing *CARenvV* we used the T = k/2r formula where T is the date of insertion, k is the divergence rate between the two LTRs and r is the neutral substitution rate of the species/lineage. For r, we utilized the previously calculated average mammalian neutral substitution rate of 2.22 x 10^−9^ [74]. Boundaries of LTRs were identified by aligning the 1000 bp surrounding the LTR35B sequences annotated by Dfam. This revealed a partial deletion in the 5’ LTR of the initially used *C*. *lupus familiaris* sequence. So, we used the *F*. *catus* CARenvV ERV LTRs for the final alignment and age calculation. We calculated the divergence (k) between 5’ and 3’ LTRs of this ERV for *F*. *catus* using the Tamura-Nei model [112] as implemented in MEGA 11 [113]. The alignment of the 5’ and 3’ LTRs is shown in S1 File.

### Analysis of RNA sequencing data

For the *in-silico* analysis of the RNA sequencing data from *B*. *taurus* and *C*. *lupus familiaris* tissues, FastQ files were downloaded from the following projects at the NCBI Sequence Read Archive (SRA) database: *C*. *lupus familiaris tissues*; PRJNA396033, *C*. *lupus familiaris* placenta: PRJNA314981, *C*.*lupus familiaris* ovary and testes: PRJNA78827, *B*. *taurus* tissues: PRJNA379574, PRJNA177791, *B*. *taurus* placenta: PRJNA314981. Raw reads were trimmed using Trimmomatic v0.38 [3] and the quality of the trimmed reads was checked using FastQC (http://www.bioinformatics.babraham.ac.uk/projects/fastqc/). HISAT2 v2.1.0 [114] was used to align the trimmed reads to the *C*. *lupus familiaris* (GCF_014441545.1) or *B*. *taurus* genome assembly (GCF_002263795.1) using Refseq annotation files. Featurecounts was used together with these annotation files to obtain read counts of each gene [115]. DEseq2 [116] as implemented in the Galaxy platform [117] with default parameters was used to normalize the read count data obtained from featurecounts for each gene. In addition to *CARenvV* and *ARTenvV*, read count data was analyzed for a liver specific gene (*VTN*) and a housekeeping gene (*GAPDH*) for each species to control for mapping and normalization accuracy.

### Maximum likelihood test for detecting positive selection

To test for codon evolution in *CARenvV* and *ARTenvV* ORF sequences, we used codeml of PAML 4.9 [72] in addition to two programs on the DataMonkey webserver: MEME, and FEL [73]. Separately aligned *CARenvV* and *ARTenvV* ORF sequences from 45 and 68 species respectively were manually inspected to exclude any indels that occurred in more than a few species. Likelihood ratio tests were performed to compare a pair of site-specific models: M8, a positive-selection model with beta distribution *dN*/*dS* values was compared to M7, another neutral model with beta distribution that does not allow positive selection. Chi-square analysis was done and the model that fit the data better was selected using a *p* value of 0.01. To detect the specific codons in *CARenvV* and *ARTenvV* that have evolved under positive selection, the F3x4 codon frequency model in codeml of PAML 4.9 was used, with two separate initial seed values of ω. Posterior probabilities of codons under positive selection were inferred using the BEB algorithm in the M8 model (Table 1). Alternative tests for positive-selection analyses were performed using the MEME and REL programs from DataMonkey webserver with recommended settings and *p* < 0.05.

### Simulation of *ARTenvV* and *CARenvV* sequence evolution

A Monte Carlo simulation as implemented in Seq-Gen v1.3.4 [118] was utilized to determine the probability that the *CARenvV* and *ARTenvV* genes would retain an intact ORF under neutral evolution over 54 and 64 million years, respectively. Ancestral sequences for *CARenvV* and *ARTenvV* were inferred using PAML4 [72] and full length orthologs of *CARenvV* and *ARTenvV*. The mammalian neutral rate of evolution of 2.2 × 10^−9^ [74] was used to calculate the branch lengths of the trees used in Seq-Gen. Simulated sequences that contained a premature stop codon and/or a mutation in the start codon were counted for 100,000 iterations of the simulation. The transition/ transversion ratio was assumed to be 4.

## Supporting information

S1 File Pairwise alignment of 5’ and 3’ LTRs of *F*. *catus CARenvV* ERV. (TXT)

S2 File Amino acid alignment of the Env protein of endogenous and exogenous retroviruses. (TXT)

S3 File Amino acid alignment of the RTase domains of endogenous and exogenous retroviruses. (TXT)

S4 File Pairwise nucleotide identity matrix for *CARenvV* and *ARTenvV*. (CSV)

S5 File Pairwise nucleotide identity and *dN/dS* values for *CARenvV*. Pairwise percentage nucleotide identity (lower triangle) and the pairwise *dN/dS* values (upper triangle) of *CARenvV* ORF of the indicated species of Carnivora are shown.
(XLSX)

S6 File Pairwise nucleotide identity and *dN/dS* values for *ARTenvV*. Pairwise percentage nucleotide identity (lower triangle) and the pairwise *dN/dS* values (upper triangle) of *ARTenvV* ORF of the indicated species of Artiodactyla are shown.
(XLSX)

S7 File Sequences of full length *ARTenvV* and *CARenvV* mRNA from 12 species of Artiodactyla and Carnivora. (TXT)

S1 Fig Pairwise alignment, repeat annotation and nucleotide identity of *ARTenvV* ERVs from *B*. *taurus* and *C*. *dromaderius*. In blue are the repeat elements identified by the Dfam repeat database for *ARTenvV* ERV of *B*. *taurus* and *C*. *dromaderius*. In green are the computer annotated first exon of *CCNY* gene for each species. *ARTenvV* ORF of *B*. *taurus* is shown in yellow. A graph for pairwise percent nucleotide identity of each region is shown above the alignment. Dark green indicates 100% identity; green indicates >30% identity; red indicates <30% identity. This graph was generated using Geneious Prime 2020.0.5 (available at https://www.geneious.com).
(PDF)

S2 Fig Repeat annotation of the *ARTenvV* and *CARenvV* related ERVs. In orange are ERV derived repeat elements identified by the Dfam repeat database for the additional copies of *ARTenvV* and *CARenvV* related ERVs of the indicated species. Location of the ERV segments that show >75% identity to *ARTenvV* or *CARenvV* ORFs are in blue.
(PDF)

S3 Fig Conserved synteny of additional *ARTenvV* and *CARenvV* related ERVs. Genomic segments containing the **A.** *ARTenvV* or **B.** *CARenvV* related additional ERVs and the linked **A.** *LDLRP1B* or **B.** *SPTLC3* genes were extracted from the NCBI genome database for each of the indicated species and aligned using the MultiPipMaker alignment tool. Location of the **A.** *ARTenvV* or **B.** *CARenvV* related ERVs are shown in black boxes in the **A.** *B*. *taurus* or **B.** *C*. *lupus familiaris* reference assemblies. Regions with more than 75% identity are shown as red boxes. Regions with less than 75% and more than 50% identity are shown as green boxes. On the left is a cladogram representing the phylogenetic relationships between the species generated using TimeTree. Starting and ending coordinates of the depicted regions of **A.** *B*. *taurus* chromosome 2 (ARS-UCD1.2) and **B.** *C*. *lupus familiaris* chromosome 24 (ROS_Cfam_1.0) are shown in red.
(PDF)

S4 Fig Genomic location and conservation of the second *ARTenvV* copy in Hippotraginae. (Upper panel) Genomic context of the second copy of *ARTenvV* is shown for the *O*. *dammah* genome (SCBI_Odam_1.1, scaffold NW_024070204.1). Refseq annotated genes are shown in green. The location of the second *ARTenvV* ORF copy is shown in black. (Lower panel) Genomic segments containing the second *ARTenvV* ORF and the *DAB2* gene were extracted from the NCBI genome database for each of the indicated species and aligned using the MultiPipMaker alignment tool [108]. Alignment of the region that immediately surrounds *ARTenvV* ORF is shown. *ARTenvV* ORF is shown in a black box in the *O*. *dammah* reference assembly. Regions with more than 75% identity are shown as red boxes. Regions with less than 75% and more than 50% identity are shown as green boxes. The cladogram on the left represents the phylogenetic relationships between the species and was generated using TimeTree [82].
(PDF)

S5 Fig Second *ARTenvV* copy contains features of LINE-1 mediated retrotransposition. Alignment of the *ARTenvV* mRNA and the region that includes the second *ARTenvV* ORF is shown for *O*. *dammah*. Exons of *ARTenvV* mRNA are annotated in red, *ARTenvV* ORF is shown in green and target site duplications (TSD) are shown in yellow.
(PDF)

S6 Fig Presence of the *ARTenvV* retroduplicated copy in *O*. *dammah*. Upper panel shows the *ARTenvV* retroduplicated copy in the *O*. *dammah* genome with PCR primers indicated with arrows. The *ARTenvV* ORF is shown in yellow and the Refseq annotated gene in green. Lower panel shows the PCR products of the indicated species. The gel is representative of two independent experiments.
(PDF)

S7 Fig Phylogeny of *ARTenvV*. The *ARTenvV* ORF was aligned from the indicated species of Artiodactyla and a maximum likelihood phylogenetic tree was generated via RaxML with 500 replicates. Bootstrap values that are below 70 are shown at the indicated nodes. Two infraorder classifications are shown on the right. Scale bar represents 0.02 nucleotide substitutions per site.
(PDF)

S8 Fig Phylogeny of *CARenvV*. *CARenvV* ORF was aligned from the indicated species of Carnivora and a maximum likelihood phylogenetic tree was generated via RaxML with 500 replicates. Bootstrap values that are below 70 are shown at the indicated nodes. Phylogenetic classifications for suborders of Carnivora are provided on the right. Scale bar represents 0.1 nucleotide substitutions per site.
(PDF)

S9 Fig Characterization of the transcripts encoding *ARTenvV*. Schematic of the genomic region that contains the annotated mRNA of *B*. *taurus ARTenvV* with the sequence of the indicated regions shown below. Alignment of the 5’ and 3’ end of the mRNA for the indicated species obtained via RACE-PCR is shown in the lower panel. Locations of the splice donor and acceptor sites, predicted poly A signal, and GT rich regions that follow the end of the transcripts are indicated.
(PDF)

S10 Fig Characterization of the transcripts encoding for *CARenvV*. Schematic of the genomic region that contains the annotated mRNA of *F*. *catus CARenvV* with the sequence of the indicated regions shown below. The alignment of the 5’ and 3’ end of the mRNA for the indicated species obtained via RACE-PCR is shown in the lower panel. Locations of the splice donor and acceptor sites, predicted poly A signal and T rich regions that follow the end of the transcripts are indicated.
(PDF)

S1 Table Genome assemblies used in this study. (DOCX)

S2 Table Viral genomes and co-opted viral genes used in this study. (DOCX)

S3 Table Primers used in this study. (DOCX)
