# Supplementary figures and images for: Cross-species transmission of an ancient endogenous retrovirus and convergent co-option of its envelope gene in two mammalian orders
Source: PLoS Genet. 2022 Oct 14;18(10):e1010458. doi: 10.1371/journal.pgen.1010458 (PMC9604959; doi:10.1371/journal.pgen.1010458)

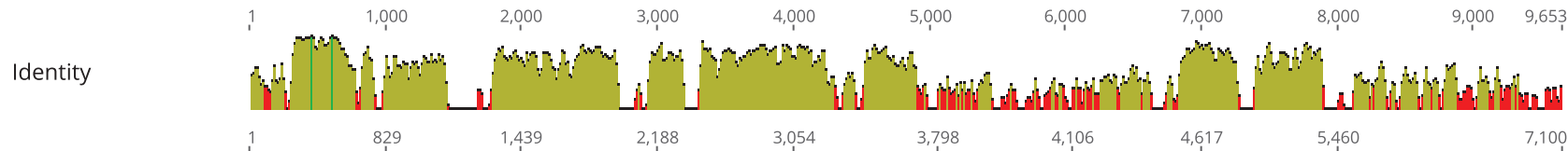

*C. dromaderius*

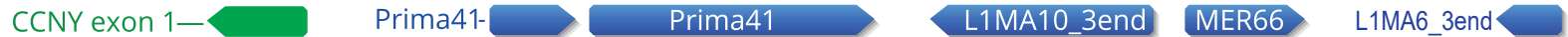

*B. taurus*

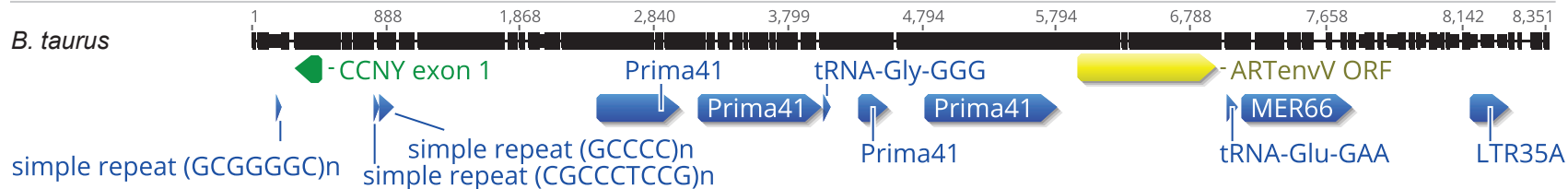

Supplement: S1 Fig — In blue are the repeat elements identified by the Dfam repeat database for ARTenvV ERV of B. taurus and C. dromaderius. In green are the computer annotated first exon of CCNY gene for each species. ARTenvV ORF of B. taurus is shown in yellow. A graph for pairwise percent nucleotide identity of each region is shown above the alignment. Dark green indicates 100% identity; green indicates >30% identity; red indicates <30% identity. This graph was generated using Geneious Prime 2020.0.5 (available at https://www.geneious.com). (PDF) [file pgen.1010458.s008.pdf]

*C. lupus familiaris*

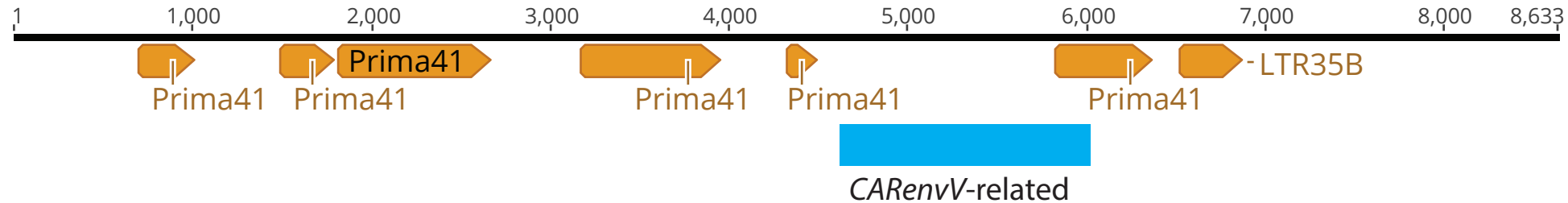

*B. taurus*

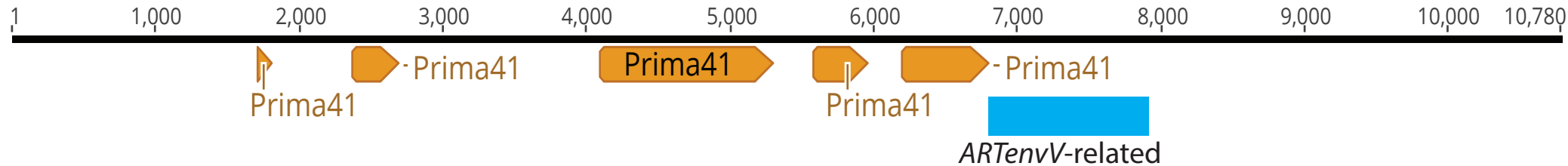

Supplement: S2 Fig — In orange are ERV derived repeat elements identified by the Dfam repeat database for the additional copies of ARTenvV and CARenvV related ERVs of the indicated species. Location of the ERV segments that show >75% identity to ARTenvV or CARenvV ORFs are in blue. (PDF) [file pgen.1010458.s009.pdf]

**A**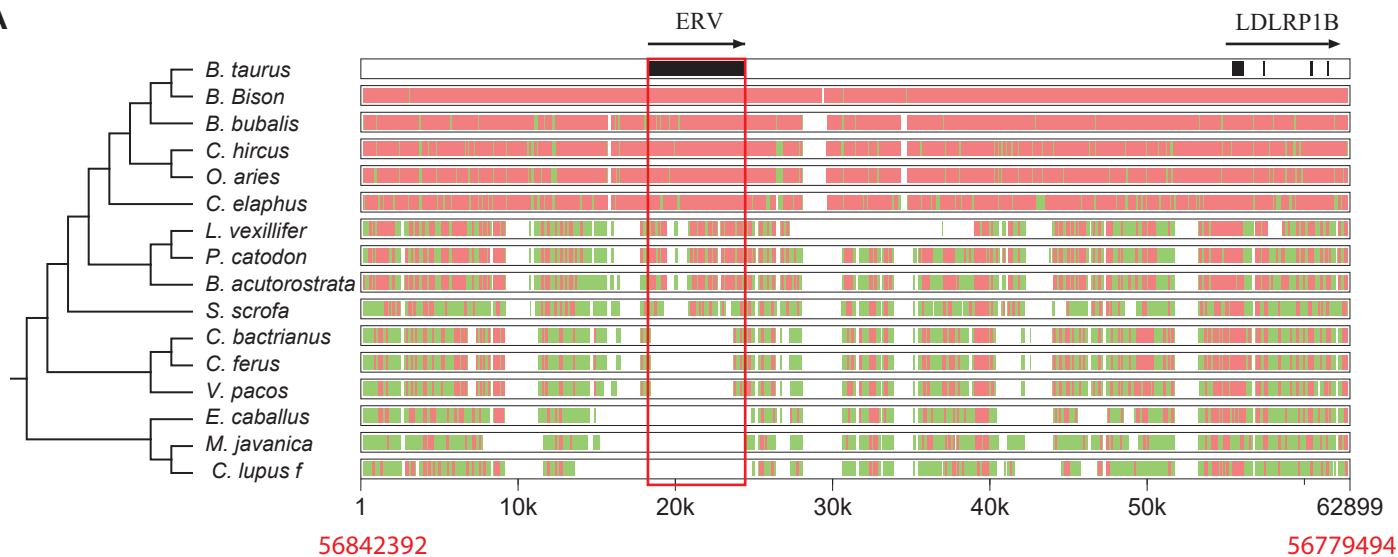**B**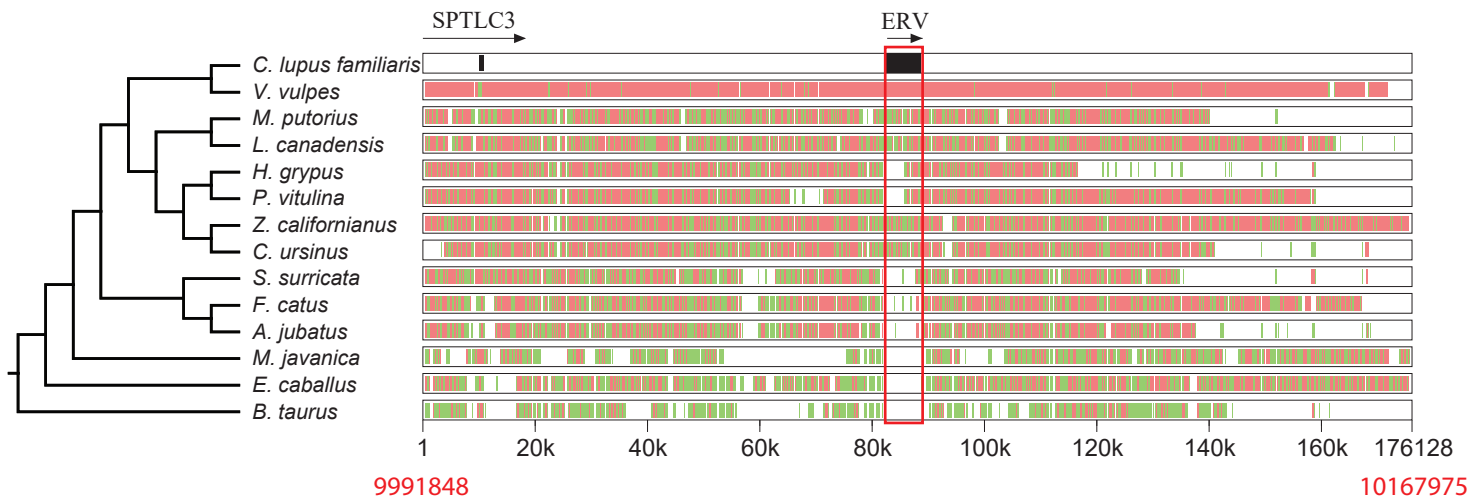

Supplement: S3 Fig — Genomic segments containing the A. ARTenvV or B. CARenvV related additional ERVs and the linked A. LDLRP1B or B. SPTLC3 genes were extracted from the NCBI genome database for each of the indicated species and aligned using the MultiPipMaker alignment tool. Location of the A. ARTenvV or B. CARenvV related ERVs are shown in black boxes in the A. B. taurus or B. C. lupus familiaris reference assemblies. Regions with more than 75% identity are shown as red boxes. Regions with less than 75% and more than 50% identity are shown as green boxes. On the left is a cladogram representing the phylogenetic relationships between the species generated using TimeTree. Starting and ending coordinates of the depicted regions of A. B. taurus chromosome 2 (ARS-UCD1.2) and B. C. lupus familiaris chromosome 24 (ROS_Cfam_1.0) are shown in red. (PDF) [file pgen.1010458.s010.pdf]

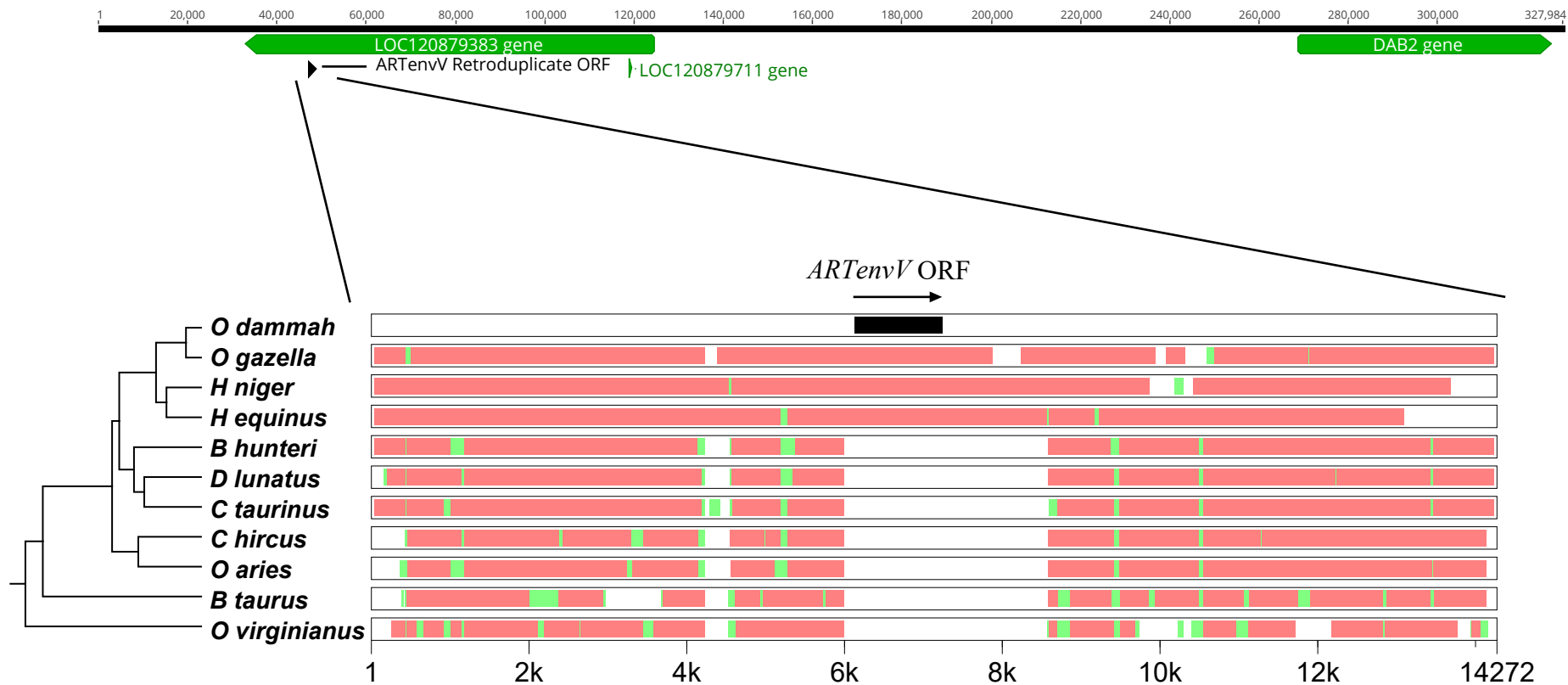

Supplement: S4 Fig — (Upper panel) Genomic context of the second copy of ARTenvV is shown for the O. dammah genome (SCBI_Odam_1.1, scaffold NW_024070204.1). Refseq annotated genes are shown in green. The location of the second ARTenvV ORF copy is shown in black. (Lower panel) Genomic segments containing the second ARTenvV ORF and the DAB2 gene were extracted from the NCBI genome database for each of the indicated species and aligned using the MultiPipMaker alignment tool [108]. Alignment of the region that immediately surrounds ARTenvV ORF is shown. ARTenvV ORF is shown in a black box in the O. dammah reference assembly. Regions with more than 75% identity are shown as red boxes. Regions with less than 75% and more than 50% identity are shown as green boxes. The cladogram on the left represents the phylogenetic relationships between the species and was generated using TimeTree [82]. (PDF) [file pgen.1010458.s011.pdf]

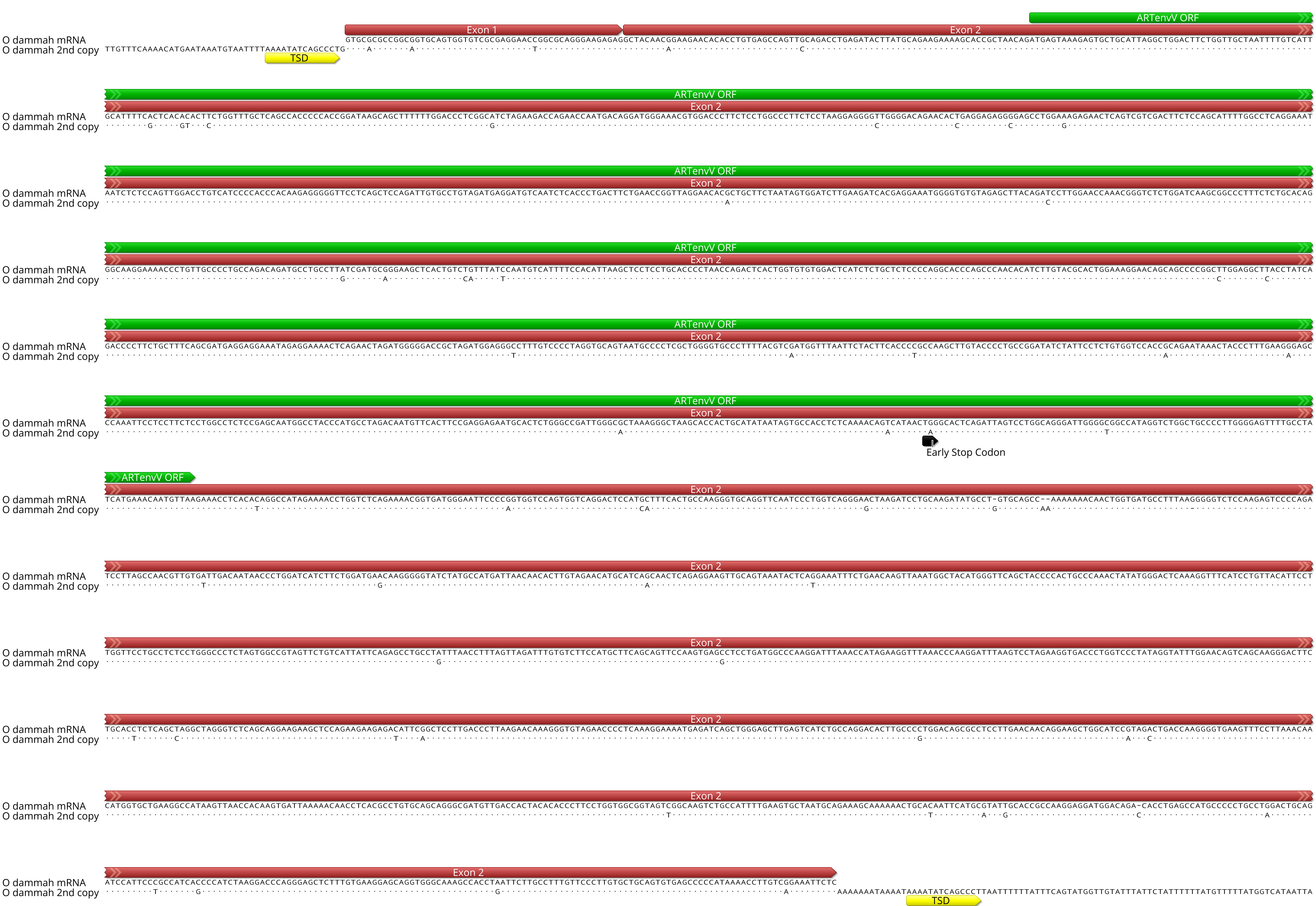

Supplement: S5 Fig — Alignment of the ARTenvV mRNA and the region that includes the second ARTenvV ORF is shown for O. dammah. Exons of ARTenvV mRNA are annotated in red, ARTenvV ORF is shown in green and target site duplications (TSD) are shown in yellow. (PDF) [file pgen.1010458.s012.pdf]

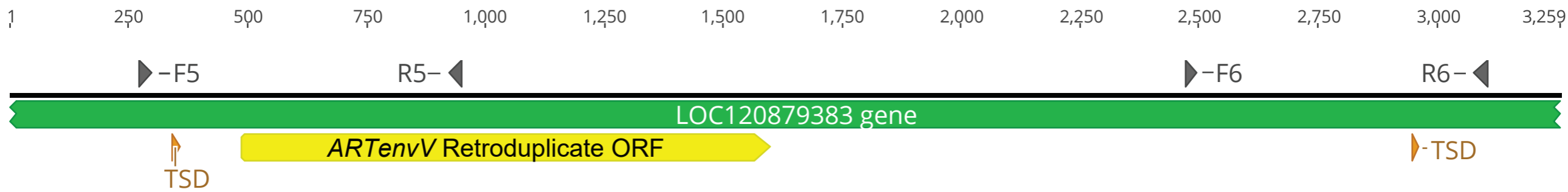

water control

*O. dammah*

*B. taurus*

*O. aries*

water control

*O. dammah*

*B. taurus*

*O. aries*

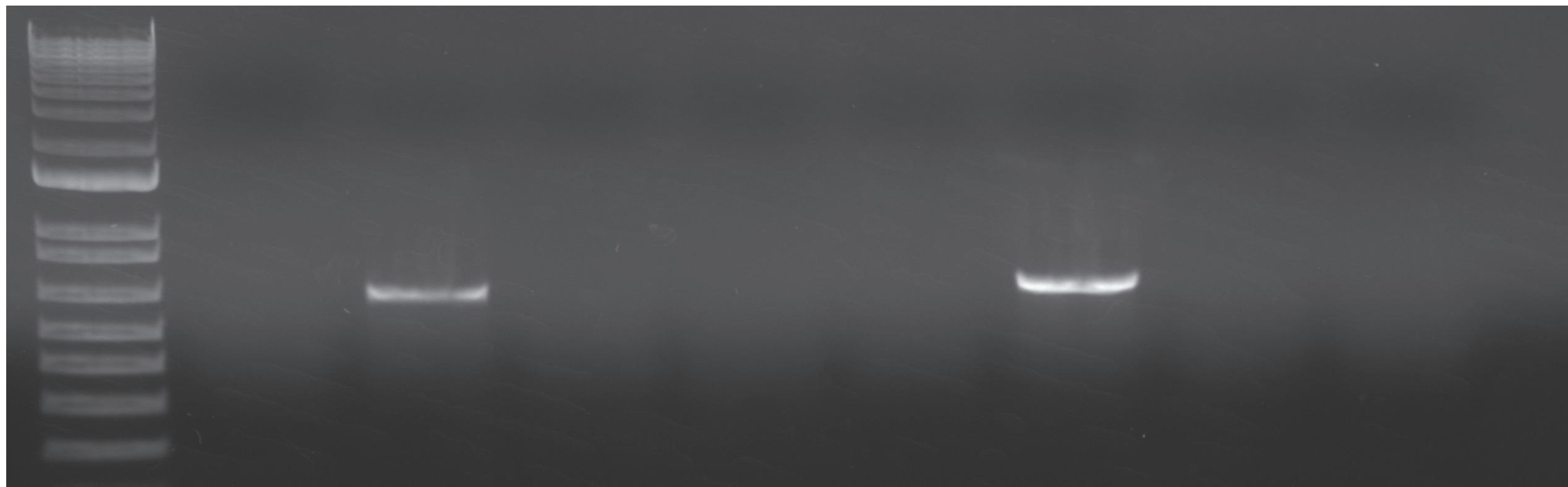

Supplement: S6 Fig — Upper panel shows the ARTenvV retroduplicated copy in the O. dammah genome with PCR primers indicated with arrows. The ARTenvV ORF is shown in yellow and the Refseq annotated gene in green. Lower panel shows the PCR products of the indicated species. The gel is representative of two independent experiments. (PDF) [file pgen.1010458.s013.pdf]

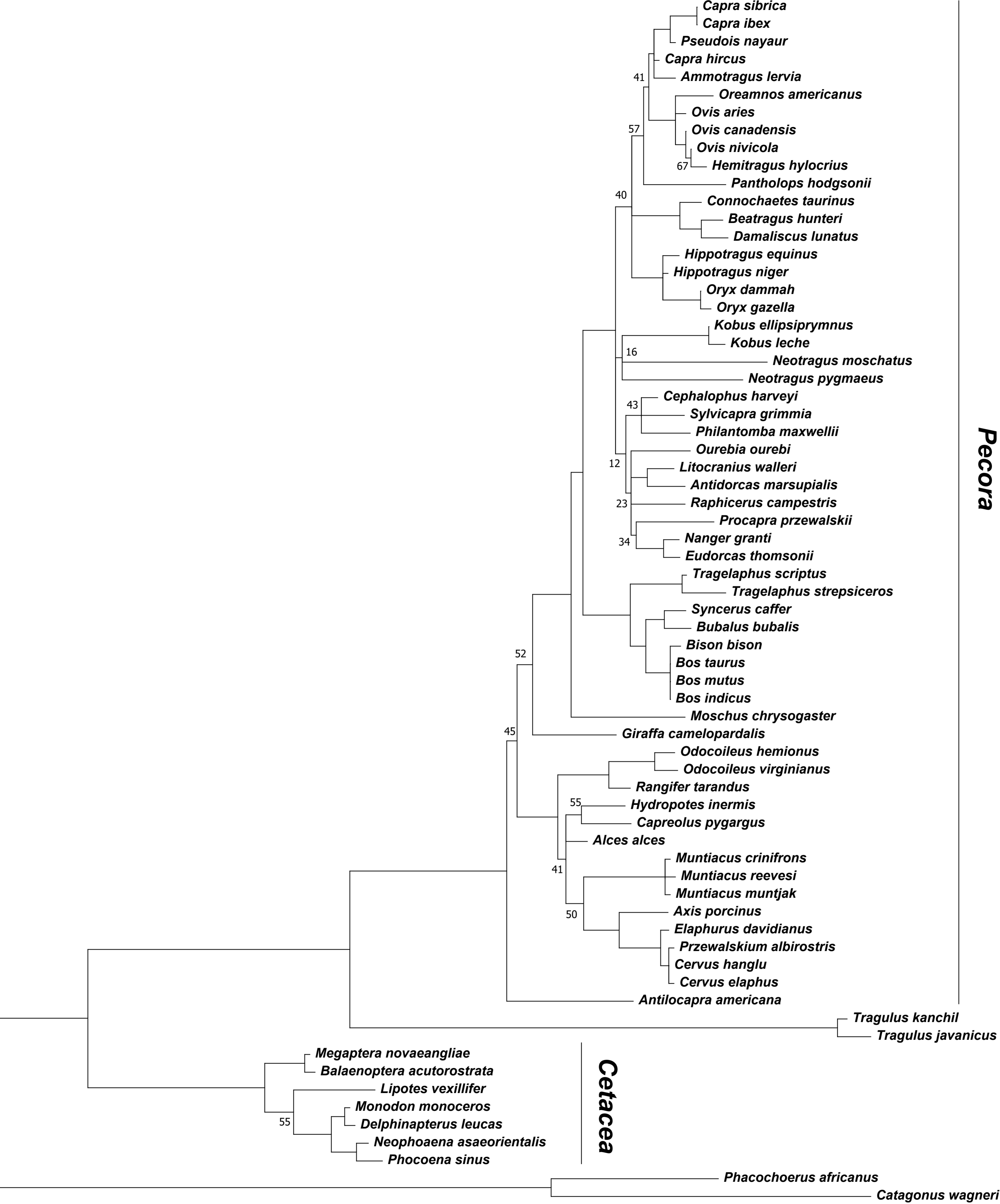

**Pecora**

**Cetacea**

0.020

Supplement: S7 Fig — The ARTenvV ORF was aligned from the indicated species of Artiodactyla and a maximum likelihood phylogenetic tree was generated via RaxML with 500 replicates. Bootstrap values that are below 70 are shown at the indicated nodes. Two infraorder classifications are shown on the right. Scale bar represents 0.02 nucleotide substitutions per site. (PDF) [file pgen.1010458.s014.pdf]

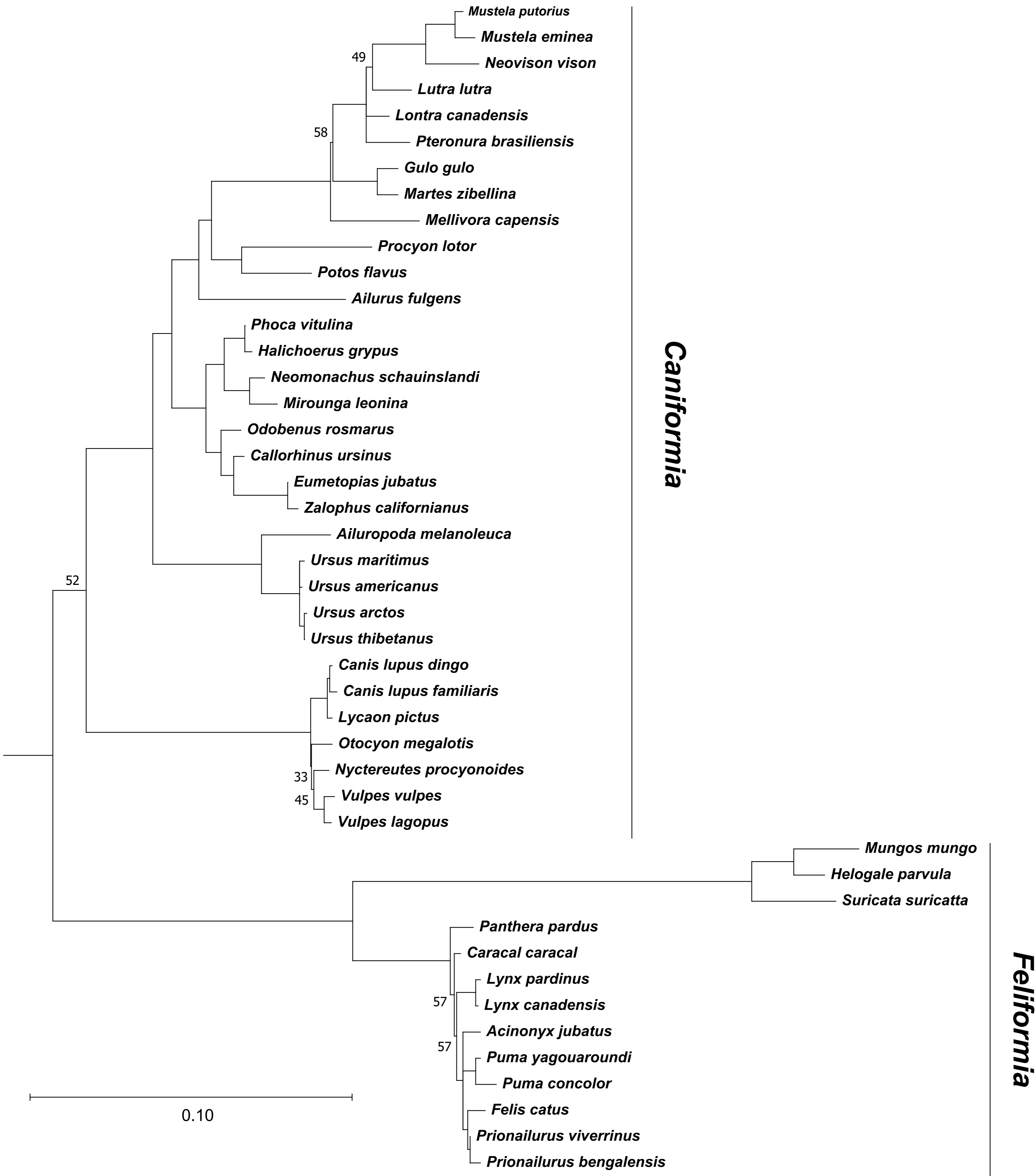

Supplement: S8 Fig — CARenvV ORF was aligned from the indicated species of Carnivora and a maximum likelihood phylogenetic tree was generated via RaxML with 500 replicates. Bootstrap values that are below 70 are shown at the indicated nodes. Phylogenetic classifications for suborders of Carnivora are provided on the right. Scale bar represents 0.1 nucleotide substitutions per site. (PDF) [file pgen.1010458.s015.pdf]

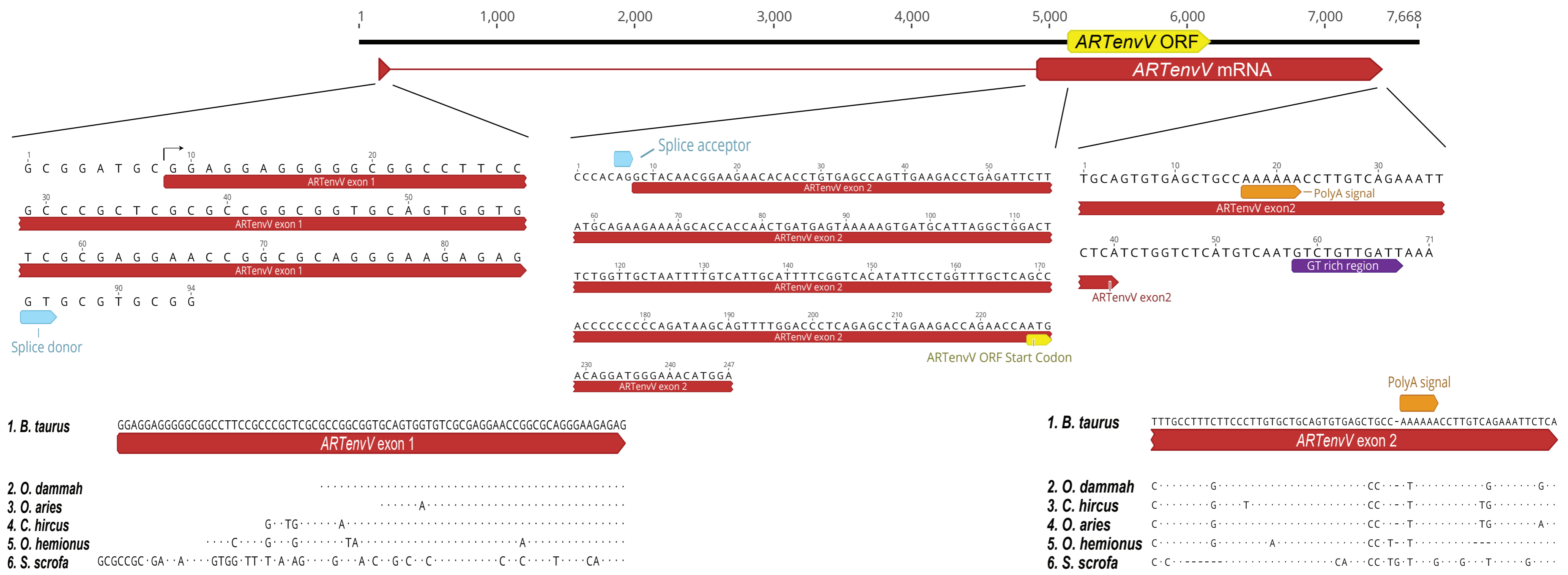

Supplement: S9 Fig — Schematic of the genomic region that contains the annotated mRNA of B. taurus ARTenvV with the sequence of the indicated regions shown below. Alignment of the 5’ and 3’ end of the mRNA for the indicated species obtained via RACE-PCR is shown in the lower panel. Locations of the splice donor and acceptor sites, predicted poly A signal, and GT rich regions that follow the end of the transcripts are indicated. (PDF) [file pgen.1010458.s016.pdf]

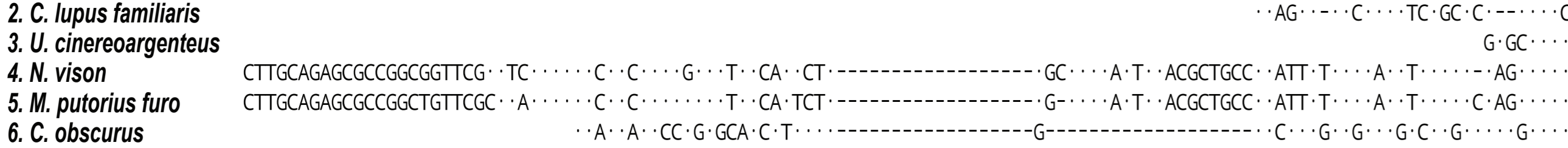

Supplement: S10 Fig — Schematic of the genomic region that contains the annotated mRNA of F. catus CARenvV with the sequence of the indicated regions shown below. The alignment of the 5’ and 3’ end of the mRNA for the indicated species obtained via RACE-PCR is shown in the lower panel. Locations of the splice donor and acceptor sites, predicted poly A signal and T rich regions that follow the end of the transcripts are indicated. (PDF) [file pgen.1010458.s017.pdf]
