# Supplementary material for: Cross-species transmission of an ancient endogenous retrovirus and convergent co-option of its envelope gene in two mammalian orders
Source: PLoS Genet. 2022 Oct 14;18(10):e1010458. doi: 10.1371/journal.pgen.1010458 (PMC9604959; doi:10.1371/journal.pgen.1010458)
Supplement: S1 Table — (DOCX) [file pgen.1010458.s018.docx]

**S1 Table. Genome assemblies used in this study**

| Species | Assembly Genbank Accession | Assembly level/scaffold N50^a^ |
| --- | --- | --- |
| **Carnivora** | | |
| Felis catus (Domestic cat) | GCF_018350175.1 | Chromosome/148,491,486 |
| Prionailurus viverrinus (fishing cat) | GCA_018119265.1 | Scaffold/3,068,410 |
| Prionailurus bengalensis (leopard cat) | GCF_016509475.1 | Chromosome/148,587,958 |
| Lynx pardinus (Spanish lynx) | GCA_900661375.1 | Scaffold/1,519,745 |
| Lynx canadensis (Canada lynx) | GCF_007474595.2 | Chromosome/147,275,701 |
| Puma yagouaroundi (jaguarundi) | GCF_014898765.1 | Scaffold/49,273,425 |
| Puma concolor (puma) | GCF_003327715.1 | Scaffold/100,532,876 |
| Caracal caracal (caracal) | GCA_016801355.1 | Scaffold/2,085,423 |
| Acinonyx jubatus (cheetah) | GCF_003709585.1 | Scaffold/48,500,042 |
| Panthera pardus (leopard) | GCF_001857705.1 | Scaffold/21,701,857 |
| Panthera tigris (tiger) | GCF_018350195.1 | Chromosome/146,942,463 |
| Paradoxurus hermaphroditus (Asian palm civet) | GCA_004024585.1 | Scaffold/71,823 |
| Hyaena hyaena (striped hyena) | GCF_003009895.1 | Scaffold/2,001,327 |
| Crocuta crocuta (spotted hyena) | GCA_008692635.1 | Scaffold/7,236,831 |
| Proteles cristatus (Aardwolf) | GCA_017311185.1 | Scaffold/1,308,801 |
| Cryptoprocta ferox (fossa) | GCA_004023885.1 | Scaffold/173,473 |
| Helogale parvula (dwarf mongoose) | GCA_004023845.1 | Scaffold/179,119 |
| Suricata suricatta (meerkat) | GCF_006229205.1 | Chromosome/141,453,419 |
| Mungos mungo (banded mongoose) | GCA_004023785.1 | Scaffold/236,501 |
| Canis lupus familiaris (domestic dog) | GCF_014441545.1 | Chromosome/64,037,277 |
| Lycaon pictus (African hunting dog) | GCA_004216515.1 | Scaffold/7,494,581 |
| Nyctereutes procyonoides (raccoon dog) | GCA_905146905.1 | Scaffold/53,959,811 |
| Otocyon megalotis (bat-eared fox) | GCA_017311455.1 | Scaffold/728,073 |
| Vulpes vulpes (red fox) | GCF_003160815.1 | Scaffold/12,472,085 |
| Vulpes lagopus (Arctic fox) | GCF_018345385.1 | Chromosome/131,537,142 |
| Ursus maritimus (polar bear) | GCF_017311325.1 | Scaffold/72,237,886 |
| Ursus arctos (brown bear) | GCF_003584765.2 | Chromosome/36,708,181 |
| Ursus americanus (American black bear) | GCA_003344425.1 | Scaffold/189,900 |
| Ursus thibetanus (Asian black bear) | GCA_009660055.1 | Scaffold/26,803,000 |
| Ailuropoda melanoleuca (giant panda) | GCF_002007445.1 | Chromosome/129,245,720 |
| Halichoerus grypus (gray seal) | GCF_012393455.1 | Scaffold/1,033,864 |
| Phoca vitulina (harbor seal) | GCF_004348235.1 | Scaffold/41,024,070 |
| Neomonachus schauinslandi (Hawaiian monk seal) | GCF_002201575.2 | Chromosome/150,810,825 |
| Mirounga leonine (Southern elephant seal) | GCF_011800145.1 | Scaffold/54,232,831 |
| Odobenus rosmarus divergens (Pacific walrus) | GCF_000321225.1 | Scaffold/2,616,778 |
| Callorhinus ursinus (northern fur seal) | GCF_003265705.1 | Scaffold/31,506,801 |
| Eumetopias jubatus (Steller sea lion) | GCF_004028035.1 | Scaffold/14,018,600 |
| Zalophus californianus (California sea lion) | GCF_009762305.2 | Chromosome/147,124,152 |
| Ailurus fulgens (red panda) | GCA_002007465.1 | Scaffold/2,983,736 |
| Procyon lotor (raccoon) | GCA_015708975.1 | Scaffold/1,449,398 |
| Potos flavus (kinkajou) | GCA_015708855.1 | Scaffold/3,555,714 |
| Mellivora capensis (ratel) | GCA_004024625.1 | Scaffold/59,143 |
| Gulo gulo (wolverine) | GCA_900006375.2 | Scaffold/178,272 |
| Martes zibellina (sable) | GCA_012583365.1 | Scaffold/5,199,373 |
| Mustela erminea (ermine) | GCF_009829155.1 | Chromosome/130,149,454 |
| Mustela putorius furo (domestic ferret) | GCF_011764305.1 | Scaffold/23,598,655 |
| Neogale vison (American mink) | GCF_020171115.1 | Chromosome/220,349,319 |
| Pteronura brasiliensis (giant otter) | GCA_004024605.1 | Scaffold/119,023 |
| Lontra canadensis (Northern American river otter) | GCF_010015895.1 | Scaffold/18,460,785 |
| Enhydra lutris (sea otter) | GCF_002288905.1 | Scaffold/38,751,465 |
| Lutra lutra (Eurasian river otter) | GCA_902655055.2 | Chromosome/149,004,807 |
| **Pholidota** | | |
| Manis javanica (Malayan pangolin) | GCF_014570535.1 | Scaffold/13,854,118 |
| **Perissodactyla** | | |
| Equus caballus (horse) | GCF_002863925.1 | Chromosome/87,230,776 |
| **Chiroptera** | | |
| Rhinolophus ferrumequinum (greater horseshoe bat) | GCA_004115265.3 | Chromosome/88,025,743 |
| **Eulipotyphla** | | |
| Talpa occidentalis (Iberian mole) | GCF_014898055.1 | Scaffold/119,794,413 |
| **Artiodactyla** | | |
| Bos taurus (cattle) | GCF_002263795.1 | Chromosome/103,308,737 |
| Bos indicus (zebu cattle) | GCF_000247795.1 | Chromosome/106,310,653 |
| Bos mutus (wild yak) | GCF_000298355.1 | Scaffold/1,407,960 |
| Bison bison (American bison) | GCF_000754665.1 | Scaffold/7,192,658 |
| Syncerus caffer (African buffalo) | GCA_902825105.1 | Scaffold/69,160,875 |
| Bubalus bubalis (water buffalo) | GCF_019923935.1 | Chromosome/116,997,125 |
| Tragelaphus strepsiceros (greater kudu) | GCA_006410795.1 | Scaffold/511,483 |
| Tragelaphus scriptus (bushbuck) | GCA_006410495.1 | Scaffold/890,554 |
| Hippotragus equinus (roan antelope) | GCA_016433095.1 | Scaffold/8,357,620 |
| Hippotragus niger (sable antelope) | GCA_006942125.1 | Scaffold/4,586,323 |
| Oryx gazella (gemsbok) | GCA_003945745.1 | Scaffold/1,579,191 |
| Oryx dammah (scimitar-horned oryx) | GCF_014754425.2 | Scaffold/100,398,400 |
| Beatragus hunter (hirola) | GCA_004027495.1 | Scaffold/69,303 |
| Damaliscus lunatus (topi) | GCA_006408505.1 | Scaffold/1,166,796 |
| Connochaetes taurinus (brindled gnu) | GCA_006408615.1 | Scaffold/366,224 |
| Oreamnos americanus (mountain goat) | GCA_009758055.1 | Scaffold/66,616,962 |
| Ammotragus lervia (aoudad) | GCA_002201775.1 | Scaffold/1,301,762 |
| Hemitragus hylocrius (Nilgiri tahr) | GCA_004026825.1 | Scaffold/85,340 |
| Capra hircus (goat) | GCF_001704415.1 | Chromosome/87,277,232 |
| Capra ibex (Alpine ibex) | GCA_006410555.1 | Scaffold/61,905,114 |
| Capra sibirica (Siberian ibex) | GCA_003182615.2 | Scaffold/15,190,720 |
| Pseudois nayaur (bharal) | GCA_003182575.1 | Scaffold/21,385 |
| Ovis aries (sheep) | GCF_016772045.1 | Chromosome/101,274,418 |
| Ovis canadensis (bighorn sheep) | GCA_004026945.1 | Scaffold/69,397 |
| Ovis nivicola (snow sheep) | GCA_903231385.1 | Scaffold/2,011,554 |
| Philantomba maxwellii (Maxwell's duiker) | GCA_006410695.1 | Scaffold/383,899 |
| Sylvicapra grimmia (bush duiker) | GCA_006408735.1 | Scaffold/583,330 |
| Cephalophus harveyi (Harvey's duiker) | GCA_006410635.1 | Scaffold/365,466 |
| Pantholops hodgsonii (chiru) | GCA_000400835.1 | Scaffold/2,772,860 |
| Litocranius walleri (gerenuk) | GCA_006410535.1 | Scaffold/3,126,223 |
| Antidorcas marsupialis (springbok) | GCA_006408585.1 | Scaffold/694,905 |
| Eudorcas thomsonii (Thomson's gazelle) | GCA_006408755.1 | Scaffold/1,581,717 |
| Nanger granti (Grant's gazelle) | GCA_006408635.1 | Scaffold/528,456 |
| Ourebia ourebi (oribi) | GCA_006417275.1 | Scaffold/1,259 |
| Madoqua kirkii (Kirk's dik-dik) | GCA_006408675.1 | Scaffold/27,730 |
| Raphicerus campestris (steenbok) | GCA_006410735.1 | Scaffold/537,161 |
| Procapra przewalskii (Przewalski's gazelle) | GCA_006410515.1 | Scaffold/5,522,907 |
| Neotragus moschatus (suni) | GCA_006410615.1 | Scaffold/952,090 |
| Neotragus pygmaeus (royal antelope) | GCA_006410875.1 | Scaffold/363,895 |
| Kobus ellipsiprymnus (waterbuck) | GCA_006410655.1 | Scaffold/779,552 |
| Kobus leche (lechwe) | GCA_014926565.1 | Scaffold/3,233,651 |
| Aepyceros melampus (impala) | GCA_006408695.1 | Scaffold/344,542 |
| Moschus chrysogaster (alpine musk deer) | GCA_006461725.1 | Scaffold/100,428 |
| Giraffa camelopardalis (giraffe) | GCA_017591445.1 | Chromosome/158,234,173 |
| Antilocapra americana (pronghorn) | GCA_007570785.1 | Scaffold/18,739,554 |
| Hydropotes inermis (Chinese water deer) | GCA_020226075.1 | Chromosome/74,967,728 |
| Alces alces (Eurasian elk) | GCA_007570765.1 | Scaffold/4,131,188 |
| Odocoileus hemionus (mule deer) | GCA_020976825.1 | Chromosome/72,140,960 |
| Odocoileus virginianus (white-tailed deer) | GCF_002102435.1 | Scaffold/850,721 |
| Rangifer tarandus (Reindeer) | GCA_019903745.1 | Scaffold/29,228,715 |
| Capreolus pygargus (Eastern roe deer) | GCA_012922965.1 | Scaffold/6,067,221 |
| Muntiacus crinifrons (black muntjac) | GCA_020276665.1 | Chromosome/646,574,120 |
| Muntiacus muntjak (muntjac) | GCA_008782695.1 | Chromosome/682,452,208 |
| Muntiacus reevesi (Reeves' muntjac) | GCA_020226045.1 | Chromosome/113,316,579 |
| Axis porcinus (Hog deer) | GCA_003798545.1 | Scaffold/20,764,858 |
| Cervus elaphus (red deer) | GCF_910594005.1 | Chromosome/83,473,711 |
| Cervus hanglu (Yarkand deer) | GCA_010411085.1 | Chromosome/77,688,133 |
| Przewalskium albirostris (white-lipped deer) | GCA_006408465.1 | Scaffold/3,769,372 |
| Elaphurus davidianus (Pere David's deer) | GCA_021018665.1 | Scaffold/3,039,716 |
| Tragulus javanicus (Java mouse-deer) | GCA_004024965.2 | Scaffold/14,082,842 |
| Tragulus kanchil (lesser mouse-deer) | GCA_006408655.1 | Scaffold/243,497 |
| Orcinus orca (killer whale) | GCF_000331955.2 | Scaffold/12,735,091 |
| Lagenorhynchus obliquidens (Pacific white-sided dolphin) | GCF_003676395.1 | Scaffold/28,371,583 |
| Sousa chinensis (Indo-pacific humpbacked dolphin) | GCA_007760645.1 | Scaffold/19,436,979 |
| Tursiops aduncus (Indo-pacific bottlenose dolphin) | GCA_003227395.1 | Scaffold/1,235,788 |
| Tursiops truncates (common bottlenose dolphin) | GCF_011762595.1 | Chromosome/108,430,135 |
| Globicephala melas (long-finned pilot whale) | GCF_006547405.1 | Scaffold/18,102,937 |
| Monodon monoceros (narwhal) | GCF_005190385.1 | Scaffold/107,566,389 |
| Delphinapterus leucas (beluga whale) | GCF_002288925.2 | Scaffold/31,183,418 |
| Phocoena sinus (vaquita) | GCF_008692025.1 | Chromosome/115,469,292 |
| Neophocaena asiaeorientalis (Yangtze finless porpoise) | GCF_003031525.2 | Scaffold/6,341,296 |
| Lipotes vexillifer (Yangtze River dolphin) | GCF_000442215.1 | Scaffold/2,419,148 |
| Inia geoffrensis (boutu) | GCA_004363515.1 | Scaffold/26,707 |
| Mesoplodon bidens (Sowerby's beaked whale) | GCA_004027085.1 | Scaffold/33,532 |
| Physeter catodon (sperm whale) | GCF_002837175.2 | Chromosome/122,182,240 |
| Eschrichtius robustus (grey whale) | GCA_002189225.1 | Scaffold/187,455 |
| Megaptera novaeangliae (humpback whale) | GCA_004329385.1 | Scaffold/9,138,802 |
| Balaenoptera acutorostrata (common minke whale) | GCF_000493695.1 | Scaffold/12,843,668 |
| Hippopotamus amphibius (hippopotamus) | GCA_004027065.2 | Scaffold/4,444,377 |
| Catagonus wagneri (Chacoan peccary) | GCA_004024745.2 | Scaffold/19,204,659 |
| Phacochoerus africanus (Common warthog) | GCA_016906955.1 | Chromosome/141,887,063 |
| Sus scrofa (pig) | GCF_000003025.6 | Chromosome/88,231,837 |
| Camelus ferus (Wild Bactrian camel) | GCF_009834535.1 | Chromosome/76,025,729 |
| Camelus dromedarius (Arabian camel) | GCF_000803125.2 | Chromosome/70,369,702 |
| Vicugna pacos (alpaca) | GCF_000164845.3 | Scaffold/24,022,313 |
| Lama glama (llama) | GCA_013239585.1 | Scaffold/3,269,186 |
| Lama guanicoe (guanaco) | GCA_013239625.1 | Scaffold/14,786,206 |

a Scaffold N50, length such that scaffolds of this length or longer include half the bases of the assembly
