# Supplementary material for: Cross-species transmission of an ancient endogenous retrovirus and convergent co-option of its envelope gene in two mammalian orders
Source: PLoS Genet. 2022 Oct 14;18(10):e1010458. doi: 10.1371/journal.pgen.1010458 (PMC9604959; doi:10.1371/journal.pgen.1010458)
Supplement: S3 Table — (DOCX) [file pgen.1010458.s020.docx]

**Supplementary Table 3. Primers used in this study**

| Primer Designation | Sequence | Used to amplify |
| --- | --- | --- |
| F1 | 5’ CTC TTG GCC CTT CTC CTG GGA ATG AG 3’ | CARenvV ORF |
| F2 | 5’ ATGATGGGTTGCATAATGTGGGCCC 3’ | CARenvV kusimanse ORF |
| F3 | 5’ GACTTCTGGTTGCTAATTTTGTCATTGCAT 3’ | ARTenvV ORF |
| F4 | 5’ ATATTCCTGGTTTGCTCAGCC 3’ | ARTenvV pig ORF |
| F5 | 5’ TTAGTGTGACAGACTGATATGTGGTTAT 3’ | ARTenvV retrocopy 5’ end |
| F6 | 5’ GAGTCATCTGCCAGGACACTTGCC 3’ | ARTenvV retrocopy 3’ end |
| F7 | 5’ CGT CGT TAG AAT ACC AAT AAT CCC GGC CGT GG 3’ | CARenvV cat 3’ RACE |
| F8 | 5’ TCC GCG CCT GCG CAC AGC TTC CCG AAT 3’ | CARenvV dog and gray fox 3’ RACE |
| F9 | 5’ CAG CAG TTT CCT GTG AAC TGT GGT AGC ACA AGG GTC 3’ | CARenvV Ferret and mink 3’ RACE |
| F10 | 5’ GGA GAC TTC ACG TGC CAG TGA GAC ACC GG 3’ | CARenvV *C. obscurus* 3’ RACE |
| F11 | 5’ GCA GTA GGC CGA TGT TGA CCA CTA CAC ACC CTT C 3’ | ARTenvV Cattle 3’ RACE |
| F12 | 5’ TCC TGG TGG CGG TAG TCG GCA AGT CTG CCA T 3’ | ARTenvV Sheep and goat 3’ RACE |
| F13 | 5’ GCA GCA GGG CGA TGT TGA CCA CTA CAC ACC CT 3’ | ARTenvV oryx 3’ RACE |
| F14 | 5’ CCT CAC GCC CGT GCA GTA GGG CGA TGT TGA C 3’ | ARTenvV mule deer 3’ RACE |
| F15 | 5’ GCA GCA GGG GCA GTG CTG ACC ACT GTG TTG CC 3’ | ARTenvV pig 3’ RACE |
| F16 | 5’ AAT AGG TTA GCT GTA GAT TAT CTT CTG GC 3’ | CARenvV cat 3’ UTR |
| F17 | 5’ GAA GCT GGT TTA ATT CCT CTT CAC C 3’ | CARenvV dog 3’ UTR |
| F18 | 5’ GGA GAA TGC ACT TTG GGA CGA TTG 3’ | CARenvV ferret and mink 3’ UTR |
| F19 | 5’ AGC AAC CCC TTC CTG GAG GCT C 3’ | CARenvV gray fox 3’ UTR |
| F20 | 5’ GAC AGG TTG GCT CTA GAT TAT CTT CTG G 3’ | CARenvV kusimanse 3’ UTR |
| F21 | 5’ TTC CTC TGT GGT CCA CCG CAG AAT A 3’ | ARTenvV 3’ UTR |
| F22 | 5’ GTT CCT CTG TGG TCC AGC GCA CAA C 3’ | ARTenvV pig 3’ UTR |
| F23 | 5’ GAC AGA ATT CGC CAC CAT GAT GGA ATG GAT GAA GTG GAC CC 3’ | CARenvV ORF dog pcDNA cloning |
| F24 | 5’ GAC AGA ATT CGC CAC CAT GAT GAG ACT GAT GAA GTG GAC TC 3’ | CARenvV ORF ferret and mink pcDNA cloning |
| F25 | 5’ GAC AGA ATT CGC CAC CAT GAC AGG ATG GGA AAC ATG GAC 3’ | ARTenvV ORF cow, sheep and goat pcDNA cloning |
| R1 | 5’ GAACCCATGCAACGATCTGGCTTGTCT 3’ | CARenvV ORF |
| R2 | 5’ TCAGGCAATGTTAGGCATATAAC 3’ | CARenvV kusimanse ORF |
| R3 | 5’ CCAGGCATATCTTGCAGGATCTTCGC 3’ | ARTenvV ORF |
| R4 | 5’ GAGACCAGGTTTTCTATGGCC 3’ | ARTenvV pig ORF |
| R5 | 5’ TTCCAAGGGTCTGTAAGCTCTACACAC 3’ | ARTenvV retrocopy 5’ end |
| R6 | 5’ CATGAAATTAAGCAAATAACATTAGTGCCC 3’ | ARTenvV retrocopy 3’ end |
| R7 | 5’ CCGAGGCCATGATGCTGGAGAAGTTGACAATTG 3’ | CARenvV cat 5’ RACE |
| R8 | 5’ CGT CGA GTT CTA CCA CTA GAA GTG GTA TGC TCC TA 3’ | CARenvV dog 5’ RACE |
| R9 | 5’ GGGCTGTCCTTGCTCTCGACAGAGAAGAG 3’ | CARenvV ferret 5’ RACE |
| R10 | 5’ CCA GTA GGT ACA GGA GAA GTT AGG TTT GTC ATC TTG G 3’ | CARenvV mink 5’ RACE |
| R11 | 5’ CCA GGA GAA GGG CCA AGA GGA GGG TCC ACT 3’ | CARenvV gray fox 5’ RACE |
| R12 | 5’ GGT CTG GTA GGT GAG CCT CCA GGA AGG 3’ | CARenvV kusimanse 5’ RACE |
| R13 | 5’ CAT CCT CAT CTA CCG GCA CAA TTT GGA GCT GAG GA 3’ | ARTenvV cattle 5’ RACE |
| R14 | 5’ CCT TAG GAG AAG GGC CAG GAG AAG GGT CCA TGT 3’ | ARTenvV sheep 5’ RACE |
| R15 | 5’ GGG TGA GAT TGA CAT CCT CAT CTA CAG GCA CAA TC 3’ | ARTenvV goat 5’ RACE |
| R16 | 5’ CCT TAG GAG AAG GGC CAG GAG AAG GGT CCA CGT 3’ | ARTenvV oryx 5’ RACE |
| R17 | 5’ CCT TAC GAG AAG GGC TAG GAG AAG GGT CCA TGT TTC 3’ | ARTenvV mule deer 5’ RACE |
| R18 | 5’ CTC CGT AGG AGA AGG GCC AGG AGA AGG GTC CAC C 3’ | ARTenvV pig 5’ RACE |
| R19 | 5’ CGC AGT GGA CAG AAC ATG CTT CC 3’ | CARenvV cat 3’ UTR |
| R20 | 5’ AAA GCC TGT AAA GAG GCT GAG GAG G 3’ | CARenvV dog 3’ UTR |
| R21 | 5’ CAG GCT GGT TTA GTC TGG ATT CTT C 3’ | CARenvV ferret and mink 3’ UTR |
| R22 | 5’ AGA GCC TGC AAA GAG GCT GAG GAG G 3’ | CARenvV gray fox 3’ UTR |
| R23 | 5’ CGC AGG AGG ACA GAA CAC GCT TCT 3’ | CARenvV kusimanse 3’ UTR |
| R24 | 5’ GCA GCT CAC ACT GCA GCA CAA GG 3’ | ARTenvV cattle 3’ UTR |
| R25 | 5’ CCA ACA AGG TTT TAT GGG GGC TCA CAC 3’ | ARTenvV sheep and goat 3’ UTR |
| R26 | 5’ CCG ACA AGG TTT TAT GGG GGC TCA C 3’ | ARTenvV oryx 3’ UTR |
| R27 | 5’ CCA AGG TTT TAT AGG GGC TCA CAC TG 3’ | ARTenvV mule deer 3’ UTR |
| R28 | 5’ CAG ACC AGG CTT TAT CAG GGG CTT G 3’ | ARTenvV pig 3’ UTR |
| R29 | 5’ GAC AGA TAT CGG AGT CCT GAA GAG TTT GGG GAT 3’ | CARenvV ORF dog pcDNA cloning |
| R30 | 5’ GAC AGA TAT CGG TAG AGA GCC TCT GCC TGC GT 3’ | CARenvV ORF ferret and mink pcDNA cloning |
| R31 | 5’ AAA GTC AAC ATA AAA TCC CCT TTC ATC ACC 3’ | ARTenvV ORF cow pcDNA cloning |
| R32 | 5’ AGA TTG TTT CAT GAT AGG CAA AAT TCC C 3’ | ARTenvV ORF sheep pcDNA cloning |
| R33 | 5’ AGA TTG TTT CAT CAT AGG CAA AAC TCC 3’ | ARTenvV ORF goat pcDNA cloning |
